# Assessing immune hepatotoxicity of troglitazone with a versatile liver-immune-microphysiological-system

**DOI:** 10.3389/fphar.2024.1335836

**Published:** 2024-05-30

**Authors:** Quanfeng Deng, Youlong Yang, Yuangui Liu, Mengting Zou, Guiyuan Huang, Shiqi Yang, Lingyu Li, Yueyang Qu, Yong Luo, Xiuli Zhang

**Affiliations:** ^1^ Jiangsu Key Laboratory of Neuropsychiatric Disease and College of Pharmaceutical Science, Soochow University, Suzhou, Jiangsu Province, China; ^2^ State Key Laboratory of Fine Chemicals, Department of Pharmaceutical Sciences, School of Chemical Engineering, Dalian University of Technology, Dalian, Liaoning Province, China; ^3^ Hunan Key Laboratory for Bioanalysis of Complex Matrix Samples, Changsha, Hunan Province, China

**Keywords:** microphysiological systems, hepatotoxicity, liver injury, innate immune, organ chip

## Abstract

Drug-induced liver injury is a prevalent adverse event associated with pharmaceutical agents. More significantly, there are certain drugs that present severe hepatotoxicity only during the clinical phase, consequently leading to the termination of drug development during clinical trials or the withdrawal from the market after approval. The establishment of an evaluation model that can sensitively manifest such hepatotoxicity has always been a challenging aspect in drug development. In this study, we build a liver-immune-microphysiological-system (LIMPS) to fully demonstrate the liver injury triggered by troglitazone (TGZ), a drug that was withdrawn from the market due to hepatotoxicity. Leveraging the capabilities of organ-on-chip technology allows for the dynamic modulation of cellular immune milieu, as well as the synergistic effects between drugs, hepatocytes and multiple immune cells. Through the LIMPS, we discovered that 1) TGZ can promote neutrophils to adhered hepatocytes, 2) the presence of TGZ enhances the crosstalk between macrophages and neutrophils, 3) the induction of damage in hepatocytes by TGZ at clinically relevant blood concentrations not observed in other *in vitro* experiments, 4) no hepatotoxicity was observed in LIMPS when exposed to rosiglitazone and pioglitazone, structurally similar analogs of TGZ, even at the higher multiples of blood drug concentration levels. As an immune-mediated liver toxicity assessment method, LIMPS is simple to operate and can be used to test multiple drug candidates to detect whether they will cause severe liver toxicity in clinical settings as early as possible.

## 1 Introduction

Drug-induced liver injury (DILI) is a significant adverse event associated with pharmaceutical agents (Okoi and Oda, 2020). It is particularly problematic when drugs can cause severe liver damage during the clinical phase rather than in the preclinical stage, leading to the discontinuation of drug development or withdrawal from the market. The number of drugs with this kind of DILI characteristic is only a few, but the impact is substantial for both patients and pharmaceutical companies. For example, the clinical phase III trial of TAK875, a drug used to treat diabetes, was stopped due to the occurrence of acute liver failure (Marcinak, et al., 2018). This not only resulted in a loss of investment by Takeda Pharmaceutical Company, but also raised concerns about the TAK875’s target, GPR40, which was being studied for the first time as a potential therapeutic target. It remains uncertain whether the acute liver failure is caused by the GPR40 receptor. As a precautionary measure, other candidate drugs targeting GPR40 had their clinical trials suspended.

Researchers have been striving to understand the factors that contribute to the development of severe liver injury by drugs during clinical trials (Gu, et al., 2015; Zhao, et al., 2020). The objective is to integrate these factors into preclinical assessment methods and create a new evaluation method for drug-induced liver injury (Zhang, et al., 2020; Jin, et al., 2021), which can assist drug developers to identify a more suitable candidate for clinical without hepatotoxicity. However, progress in this field has been limited thus far. In 2022, two drugs, Inarigivir (Agarwal et al., 2020; Yuen, et al., 2023) and ABI-H2158 (Agarwal et al., 2023) were suspended during Phase II trials due to their hepatotoxicities, despite demonstrating good efficacy results.

Troglitazone (TGZ), a medication that was withdrawn from the market due to liver injury, received approval for use in the US in 1997. Prior to its clinical trials, there was no indication of liver damage observed during animal experiments. However, after 3 years of use, 94 cases of severe liver failure were reported. Although not every patient treated with troglitazone developed liver injury, only a very small subgroup of patients (<0.005%) actually developed liver failure (Graham et al., 2003a). However, due to the severity of liver failure, TGZ was withdrawn from the market in 2000 (Kohlroser, et al., 2000; Chojkier, 2005). Researchers then attempted to construct various animal models to replicate severe liver damage caused by TGZ, including specific gene knockout mice and chimeric mice with a humanized liver (Ong, et al., 2007; Kakuni, et al., 2012), as well as glutathione pre-clearance models (Jia, et al., 2019). All of these models only showed transient (within 2 h) and mild but no severe liver damage. *In vitro* experiments, researchers observed that the mitochondrial function and JAK/STAT signaling pathway of liver cells were affected by TGZ on hepatocyte (Julie, et al., 2008; Rachek et al., 2009b; Jia, Oda, Tsuneyama, Urano and Yokoi, 2019), but no significant liver cell death was observed. These results cannot explain why in the reported cases of TGZ induced hepatotoxicity, some patients exhibited acute liver failure or even death (Graham et al., 2003b).

Pathological studies of TGZ-induced liver failure or death have found the presence of hepatic inflammatory infiltrates with eosinophils (Akitaka Shibuya, et al., 1998; Gitlin, et al., 1998; Murphy, et al., 2000). Recently, researchers also found that when two immuno-related proteins PD-1 and CTLA-4 were knocked out from mice simultaneously, TGZ could show a sustained ALT increase for 2 weeks (Mak, et al., 2018). Cellular experiments have demonstrated that TGZ can activate immune cells. Kim et al.’s results indicate that the TGZ-treated conditioned medium can induce an increase in pro-inflammatory factor levels (tumor necrosis factor α, interleukin 1β, interleukin 6) and activate immune cells, including Jurkat, THP-1, and NK92MI cells (Kim, et al., 2017a). All of these studies show that an immuno-environment is quite important for TGZ-induced hepatotoxicity, However, it is still unknown whether the inflammatory environment caused by the immune response can lead to severe hepatocyte damage mediated by TGZ.

The inception of liver microphysiological system (LMPS) shed a light into this issue. LMPS is a bioengineering technology that replicates the structure and functions of a human liver in a miniaturized form (Zheng, et al., 2016; Kulsharova and Kurmangaliyeva, 2021). A core advantage of the LMPS is its flexible engineering design. For instance, depending on the specific circumstances, the LMPS of various design can simulate the liver sinusoid (Nawroth, et al., 2021), liver lobule (Banaeiyan, et al., 2017), or liver zonation (Du, et al., 2021; Ya, et al., 2021). However, previous studies have not investigated liver-on-a-chip models under immune-inflammatory microenvironments, primarily due to the highly complex and intricate nature of the immune system in the human liver. It includes a variety of specialized immune cells, such as Kupffer cells, natural killer cells, and neutrophils, as well as a range of signaling molecules and cytokines that help regulate the immune response (Yao, et al., 2016). Until now, even understanding the interactions between hepatic cells and the immune system is still an ongoing area of research (Cho and Uetrecht, 2017; Uetrecht, 2019). In this study, we developed a liver microphysiological system (LMPS) that incorporates different immune cells to mimic the inflammatory environment and evaluate the hepatotoxicity induced by TGZ. Moreover, our LMPS is simple to construct and operate, making it possible as a high-throughput platform for evaluating drug candidates with DILI liabilities.

## 2 Materials and methods

### 2.1 Cells

HepG2 cells (Cat#: SCSP-510, NCACC, Shanghai, China) and HepG2-GFP (Cat#: MZ-2792, Ningbo Mingzhou Biotechnology Co., Ltd.) cells were cultured in MEM-α medium (Cat#: C12571500BT, Gibco) containing 10% FBS (Cat#: 10099-141, Gibco) supplement with 1% Penicillin–Streptomycin (Cat#: 15140122, Gibco). HL-60 cells (Cat#: CL-0110, Procell, Wuhan, China) also were cultured in IMDM medium (Cat#: C12440500BT, Gibco) containing 10% FBS supplement with 1% Penicillin–Streptomycin, and before use, they were differentiated using 1.25% DMSO (Cat#: D8418, Sigma-Aldrich) for 5 days (Referred to as dHL-60 cells in the following text). THP-1 cells (Cat#: SCSP-567, NCACC, Shanghai, China) were culture in RPMI 1640 medium (Cat#: C22400500BT, Gibco) containing 10% FBS supplement with 1% Penicillin–Streptomycin and differentiated before use by using 50 ng/mL PMA (Cat#: P8139, Sigma-Aldrich). NK-92 cells (Cat#: CL-0530, Procell, Wuhan, China) were cultured in their special medium (Cat#: CM-0530, Procell, Wuhan, China) and maintained in 5% CO_2_ at 37°C.

### 2.2 Fabrication of liver chip and cell seeding

The fabrication of the Polydimethylsiloxane (PDMS, Sylgard 184, Dow Corning, United States) layer was achieved using soft lithography employing SU-8 2075 (102001, Microchem, United States). Subsequently, the glass slide and PDMS layer surfaces underwent plasma cleaning, followed by bonding of the PDMS layer to the glass slide. A 5 μL suspension of HepG2 cells, with a concentration of 1 × 10^7^ cells/mL, was seeded into the central microchannel. Subsequently, a 50 μL suspension of THP-1 cells, at a concentration of 3 × 10^6^ cells/mL, was added (or not) to the side microchannel after adhesion of the HepG2 cells. After 1 h, 300 μL of complete medium (IMDM) were introduced into the side microchannels. On the following day, the chip’s medium was aspirated and replaced with 600 μL of medium, with or without troglitazone (Cat#: abs814605, Abixin (Shanghai) Biotechnology Co., Ltd.) in each chip. An additional 200 μL of medium was supplemented on the third day. On the fourth day, 600 μL of medium, containing 1.5 × 10^6^ cells/mL of dHL-60 cells (or NK-92cells), was introduced to the side microchannel and troglitazone was added (or not) (Supplementary Figure S9). Using DMSO to prepare a 15 mM stock solution of troglitazone, diluted 1,000 or 500 times for use.

### 2.3 Cell staining

**FIGURE 1:**
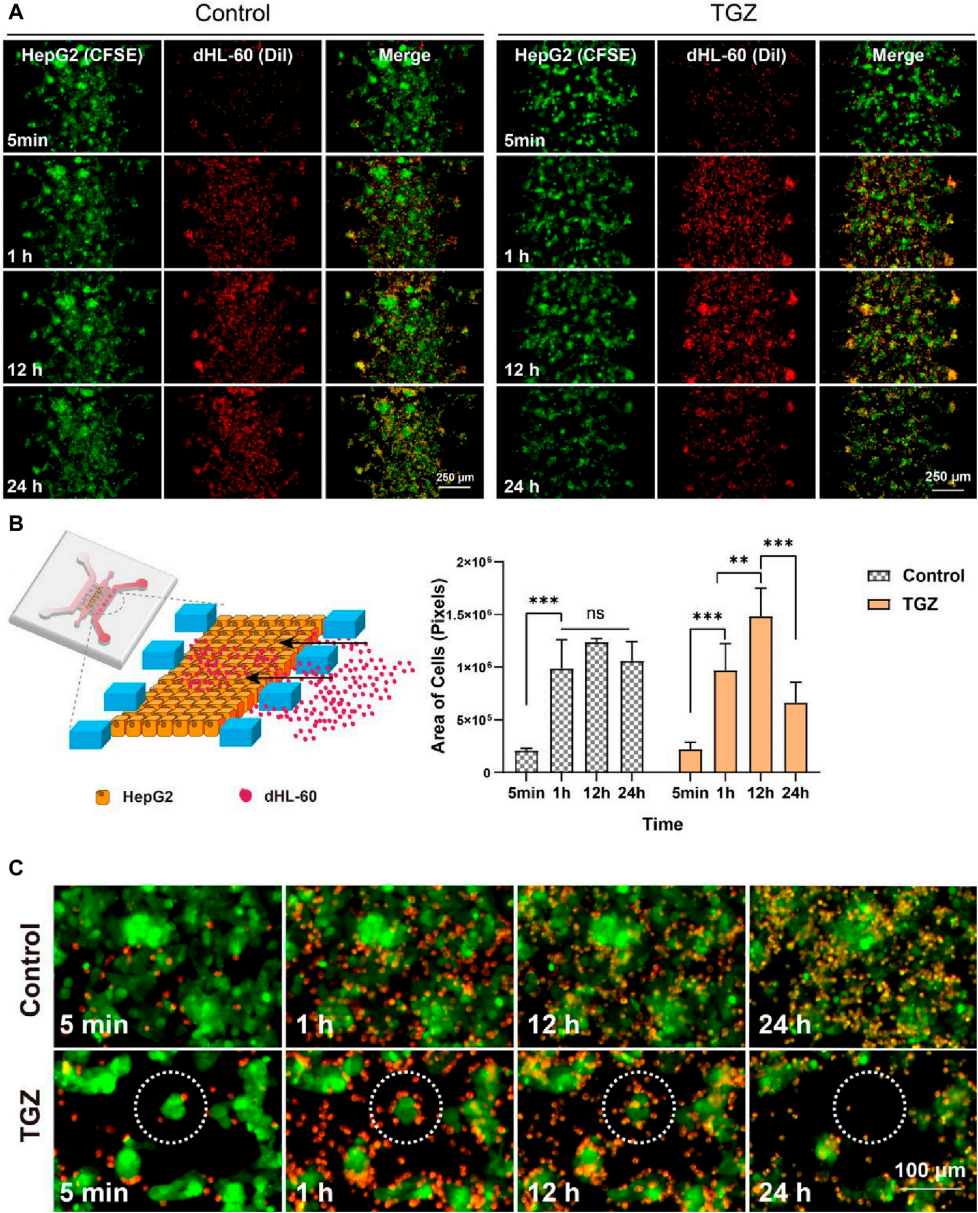
Adhesion of dHL60 cells in the LIMPS. **(A)** Adhesion of dHL-60 cells on the HepG2 cells under different circumstances. **(B)** Partial schematic diagram of the LIMPS, dynamics of the adhesion of the dHL-60 cells (*n* = 3, **p < 0.01, ***p < 0.001). **(C)** Dashed circles indicate red dHL-60 cells adhesion causing the apoptosis of green HepG2 cells.

**FIGURE 2:**
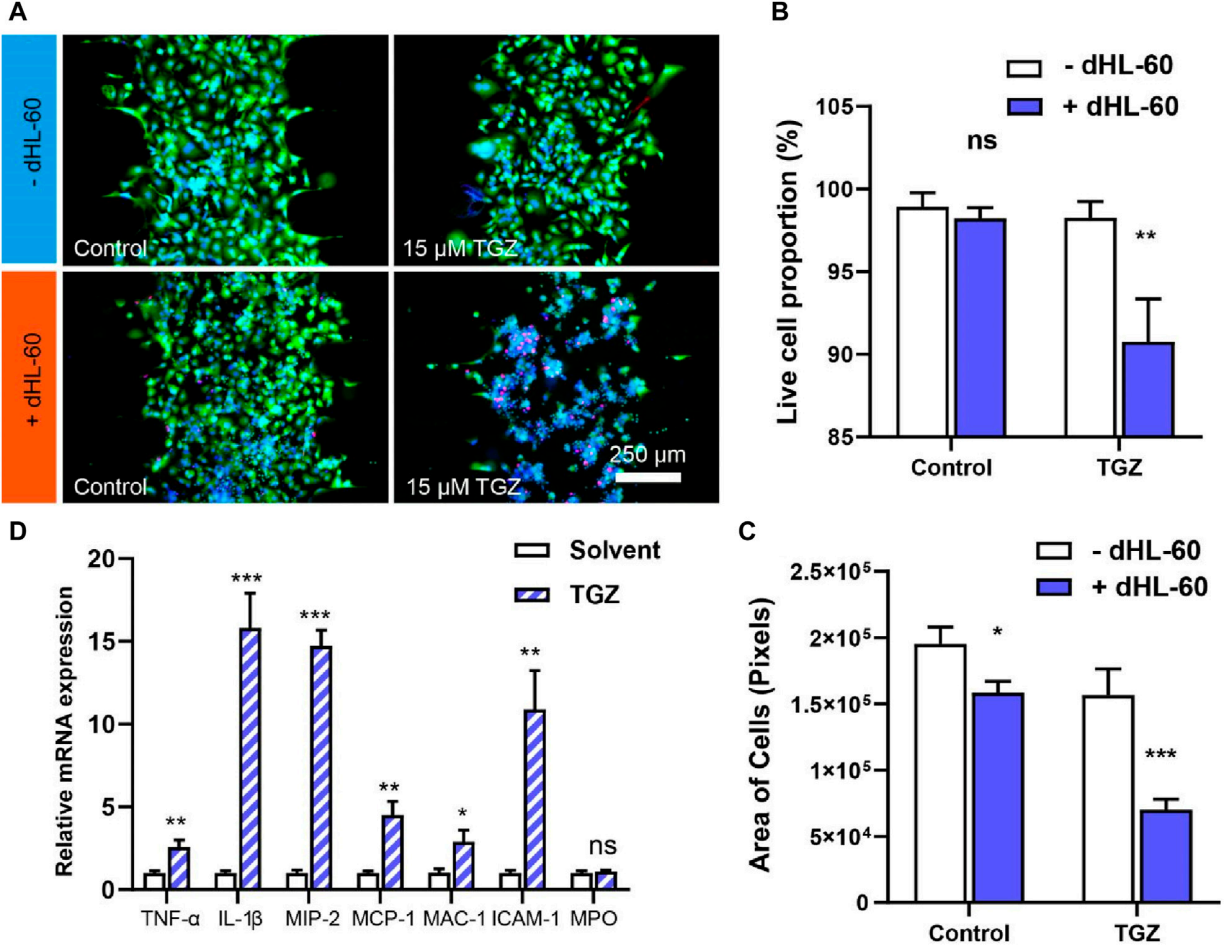
The effect of the dHL-60 cells on the hepatotoxicity due to TGZ in the LIMPS. **(A)** Live/death staining with calcein AM/PI/HOE33342 of all live cell in the LIMPS. **(B)** The relationship between the all live cell proportion and TGZ/dHL-60 cells. **(C)** The relationship between the absolute all live cell quantity and TGZ/dHL-60 cells. **(D)** The relative mRNA expression inside the dHL-60 cells (*n* = 3, *p < 0.05, **p < 0.01, ***p < 0.001)

**FIGURE 3:**
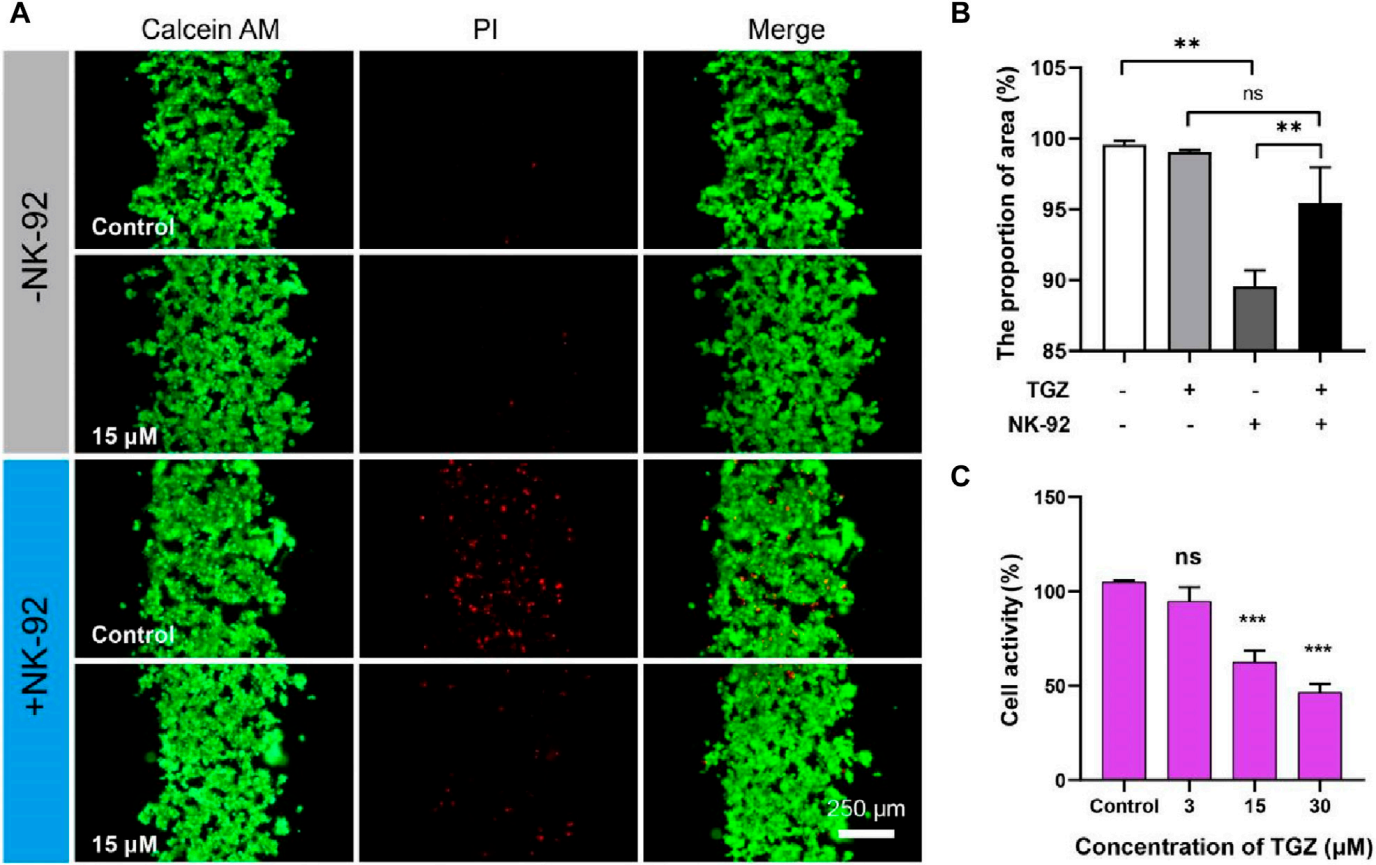
The effect of the NK-92 cells on the hepatotoxicity due to TGZ in the LIMPS. **(A)** Live/dead staining of the HepG2 cells. **(B)** Quantitative analysis of the fluorescence images in **(A)**. **(C)** Inhibitory effect of troglitazone on the NK-92 cells. (*n* = 3, *p < 0.05, **p < 0.01, ***p < 0.001).

**FIGURE 4:**
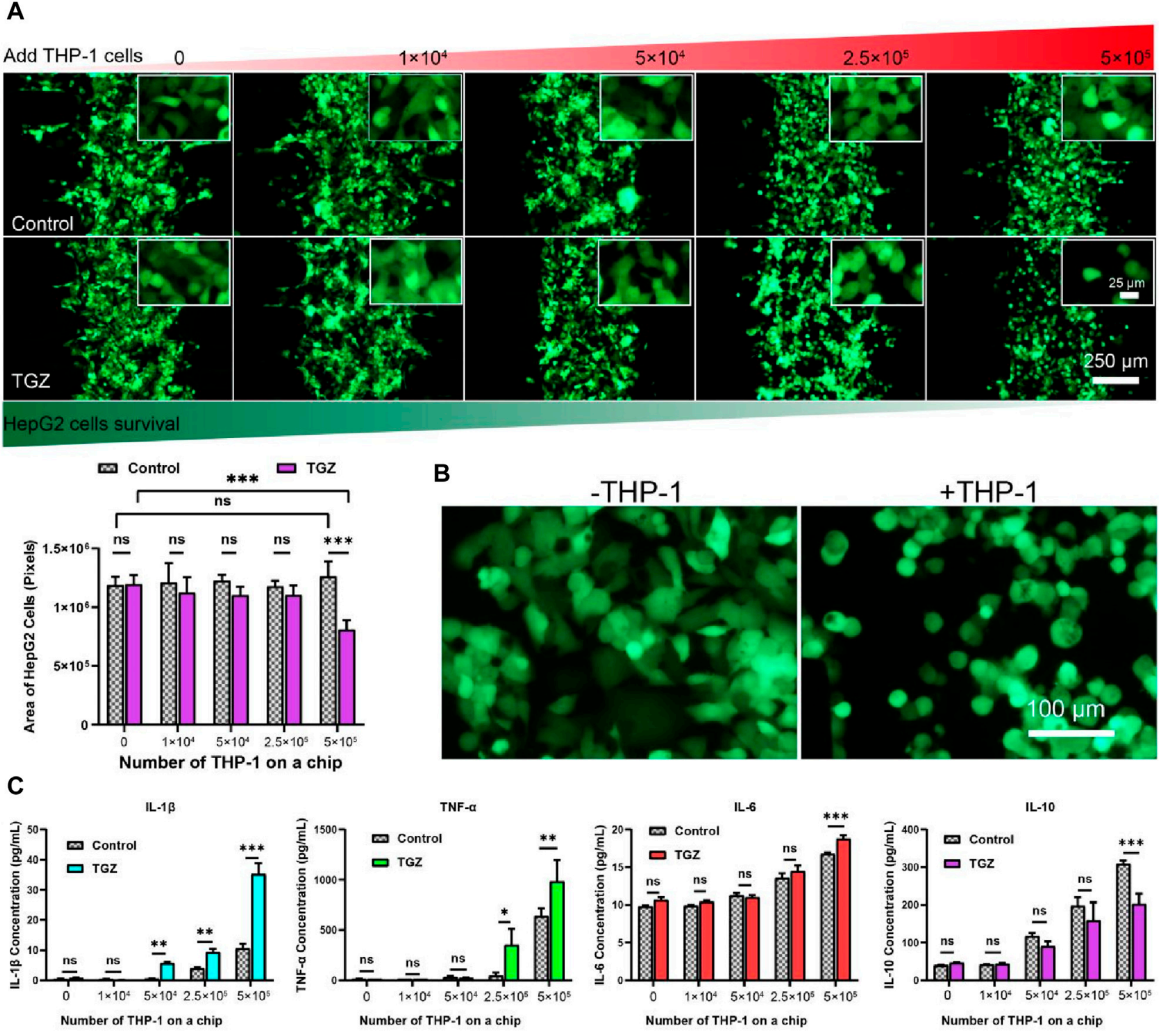
The effect of the THP-1 cells on the hepatotoxicity due to TGZ in the LIMPS. **(A)** Variation of the HepG2 cell numbers with THP-1 cell numbers with or without TGZ presence (HepG2: green, THP-1 cells not shown). **(B)** The enlarged view of the morphology of the HepG2 cells with or without the presence of THP-1 cells (5 × 105). **(C)** The secretion of the IL-1β, TNF-α, IL-6 and IL-10 with or without TGZ (*n* = 3, *p < 0.05, **p < 0.01, ***p < 0.001).

**FIGURE 5:**
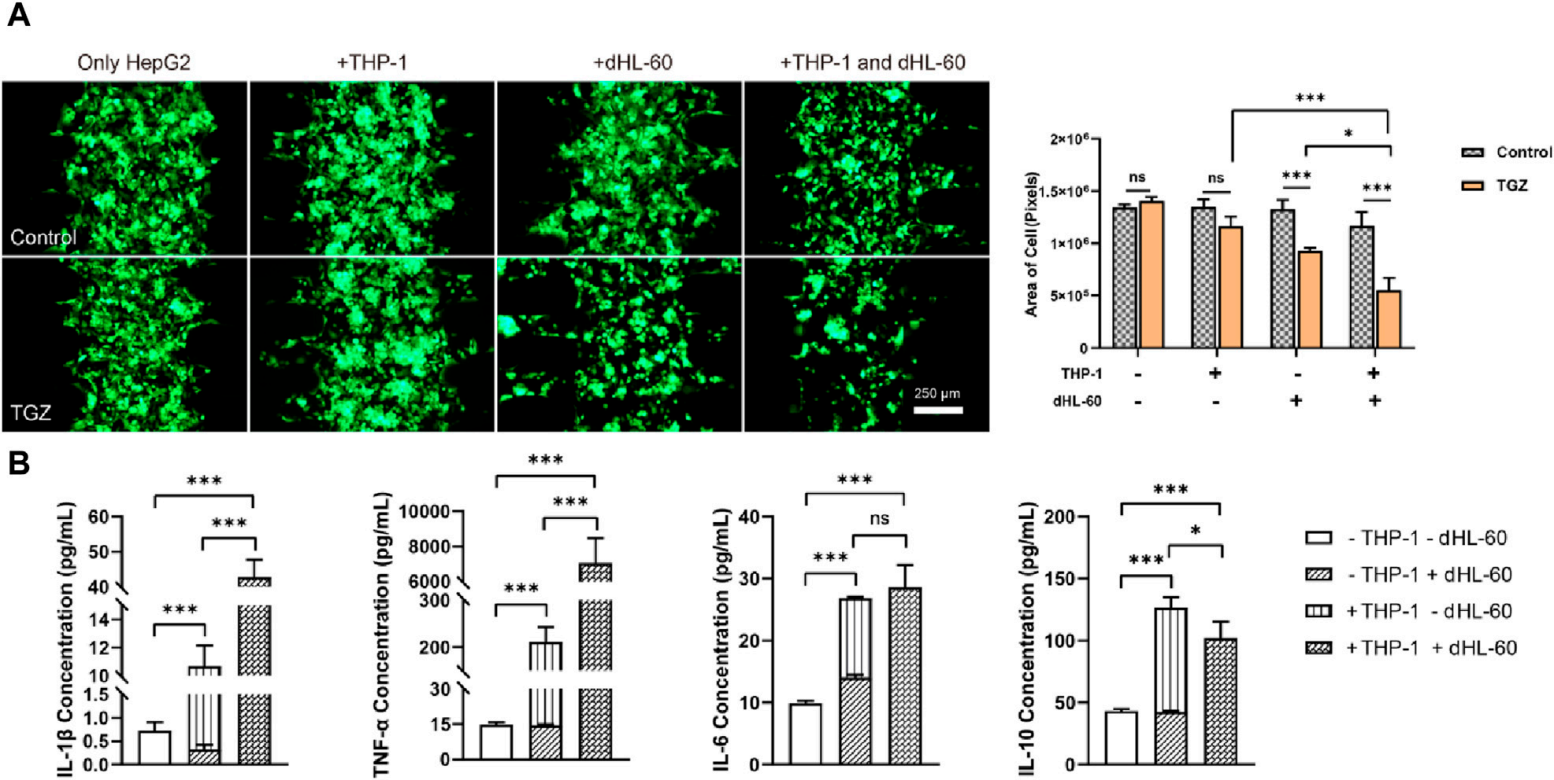
The effect of the co-presence of THP-1 and dHL-60 cells on the hepatotoxicity due to TGZ in the LIMPS. **(A)** Left: the fluorescence of HepG2-GFP with or without TGZ, THP-1 and dHL-60 cells. Right: quantitative analysis of the left images. **(B)** Secretion of the factors (IL-1β, TNF-α, IL-6, IL-10) under the different circumstances. (*n* = 3, *p < 0.05, **p < 0.01, ***p < 0.001)

**FIGURE 6:**
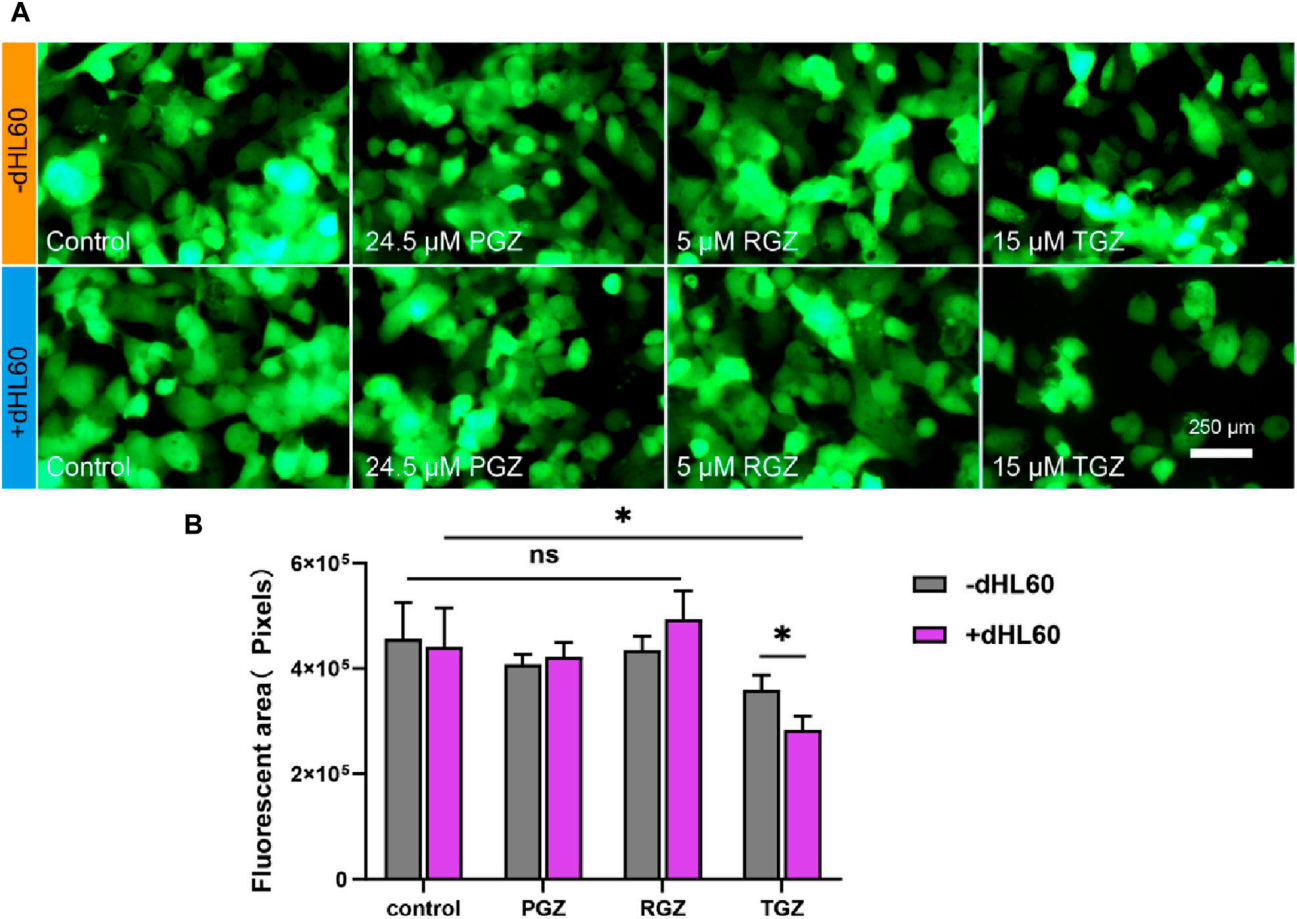
Illustrates the comparative toxicities of curcumin, pioglitazone, and rosiglitazone on the LIMPS. **(A)** Fluorescence image of cell stock in liver channels. **(B)** Quantitative analysis of the fluorescence images (*n* = 3, *p < 0.05, **p < 0.01, ***p < 0.001).

Staining of HepG2 cells with 3 μM CFSE (Cat#: abs9106, Abixin (Shanghai) Biotechnology Co., Ltd.) for 10 min at 37°C, washed three times, and then added to the chip, A 5 μL suspension of HepG2 cells, with a concentration of 1 × 107 cells/mL, was seeded into the central microchannel. After 1 h, 300 μL of complete medium were introduced into the side microchannels. On the following day, the chip’s medium was aspirated and replaced with 600 μL of medium, with or without troglitazone in each chip. An additional 200 μL of medium was supplemented on the third day. On the fourth day, Staining of dHL-60 cells with 6 μM DiL (Colored by red, Cat#: 42364, Sigma-Aldrich, DiL has 567 nm (yellow) emission.) for 20 min at 37°C, washed three times, 600 μL of medium, containing 1.5 × 106 cells/mL of dHL-60 cells, was introduced to the side microchannel and troglitazone was added (or not), and continuously observe the adhesion of HL-60 cells for 24 h (Figure 1). LIMPS with calcein AM/PI or HOE33342 followed the instruction of the corresponding commercial kits (Figures 2, 3). In addition, except for special statements, the other experiments used HepG2-GFP cells (Figures 4, 5, 6).

### 2.4 Immunocytochemistry

**FIGURE 7:**
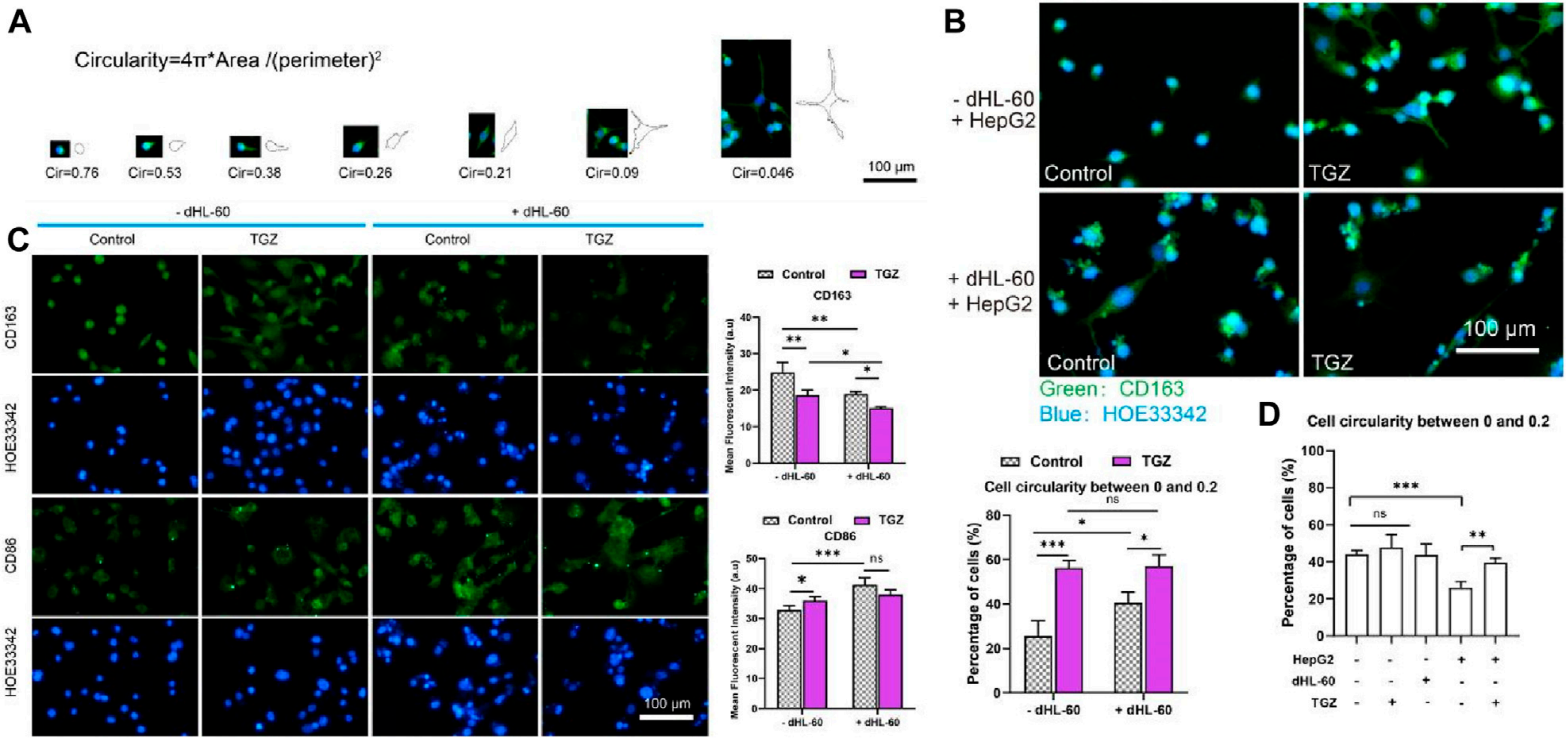
Polarization of the THP-1 cells in the LIMPS. **(A)** Evolvement of THP-1 cells under the stimulation of TGZ in the LIMPS. **(B)** The morphology of THP-1 cells under different circumstances in the side channel of the LIMPS. **(C)** CD86 and CD163 immunofluorescence of THP-1 cells under different circumstances. **(D)** Correlation between the THP-1 polarization and TGZ, HepG2 and dHL-60 cells (*n* = 3, *p < 0.05, **p < 0.01, ***p < 0.001).

At the end of each experiment (Figure 7), LIMPSs were washed three times with PBS, then fixed with a 4% paraformaldehyde solution for 5 minutes. LIMPSs were then rinsed three times with PBS and permeabilized with a 0.1% Triton X-100 solution in PBS for 5 minutes, followed by blocking with a 1% BSA solution in PBST for 1 h. LIMPSs were then incubated overnight at four degrees Celsius with the primary antibodies anti-CD163 (Cat#: 16646-1-AP, Proteintech Group, Inc.) and anti-CD86 (Cat#: 13395-1-AP, Proteintech Group, Inc.). Following three, 5-min washes with PBS, LIMPSs were incubated for 1 h at room temperature in the dark with a secondary antibody, either conjugated to Alexa-488 or Alexa-568, fluorophore. Following the secondary antibody incubation, the LIMPS surfaces were washed three times in PBS and incubated with 2 μg/mL HOE33342 for 10 min in the dark at room temperature for nuclei staining. HOE33342 solution was then removed and the cells were washed three more times with PBS. The LIMPSs were stored at four degrees Celsius until imaging. Fluorescence images were collected using an inverted fluorescence microscope (Nikon TS2R-FL, Nikon).

### 2.5 Reverse transcription quantitative polymerase chain reaction

Collecting dHL-60 cells in the LIMPS and using the RNA Isolator Total RNA Extraction Reagent R401-01 kit (Cat#: R401-01, Vazyme Biotech Co., Ltd.) to extract mRNA according to the manufacturer’s instructions. The Nanodrop spectrophotometer was used to determine the concentration and purity of mRNA. We used the HiScript III RT SuperMix for qPCR kit (Cat#: R323-01, Vazyme Biotech Co., Ltd.) to reverse transcribe mRNA into cDNA, and then used the ChamQ Universal SYBR qPCR Master Mix kit (Cat#: q711-02, Vazyme Biotech Co., Ltd.) to perform fluorescent quantitative PCR amplification of the cDNA Table 1 shows primer sequences for real-time quantitative PCR.

**TABLE 1:** Primers for quantitative real-time PCR.

Gene	Primer forward 5′-3′	Primer reverse 5′-3′
TNF-α	AGGCAGTCAGATCATCT	CTC​TGG​CAG​GGG​CTC​TTG​AT
MAC-1	TGG​TGA​AGA​CCT​ACG​AGA​AAC​TCA	CGT​AGG​TGA​CTT​TCA​GGG​TGT
MPO	CCA​TTC​AAT​GTC​ACT​GAT​GTG​CTG	CTG​TTG​TTG​CAC​ATC​CCG​GT
MIP-2	CAA​ACC​GAA​GTC​ATA​GCC​ACA​C	CCT​TCA​GGA​ACA​GCC​ACC​AA
β-actin	GGC​ACC​CAG​CAC​AAT​GAA​GA	CAT​CTG​CTG​GAA​GGT​GGA​CA
IL-1β	GCC​AGT​GAA​ATG​ATG​GCT​TAT​T	AGG​AGC​ACT​TCA​TCT​GTT​TAG​G
ICAM-1	TTC​CTC​ACC​GTG​TAC​TGG​ACT	GGG​TAA​GGT​TCT​TGC​CCA​CT

### 2.6 IL-6, IL-1β, TNF-α and IL-10 detection using ELISA

At the end of each experiment, the supernatant was collected from the LIMPS by centrifugation. The experimental procedure provided in the vendor’s protocol [Abixin (Shanghai) Biotechnology Co., Ltd.] was used. Briefly, both standard curve samples and experimental samples were loaded into precoated wells and incubated for 2 h at room temperature. All wells were then aspirated and rinsed three times with PBS. A hundred microliters of avidin-HRP were loaded into each well for 20 min at room temperature. The washing steps were repeated before the addition of one hundred microliters of tetramethylbenzidine solution to each well for 20 min at room temperature. Finally, 50 μL per well of stop solution was added, and the plates were read by a plate reader (Tecan Infinite M1000 PRO, Tecan) at a wavelength of 450 nm.

### 2.7 Statistical analysis

The data presented in graphs are the means of three separate experiments ±standard deviation. A one-way ANOVA was used to compare multiple means, followed by a Bonferroni adjustment for the number of pair-wise comparisons (utilizing GraphPad Prism 8 software). Student’s t-tests were used to compare two values with each other. A *p*-value of <0.05 was considered significant.

## 3 Results

### 3.1 Liver-immune-microphysiological-system (LIMPS)

**FIGURE 8:**
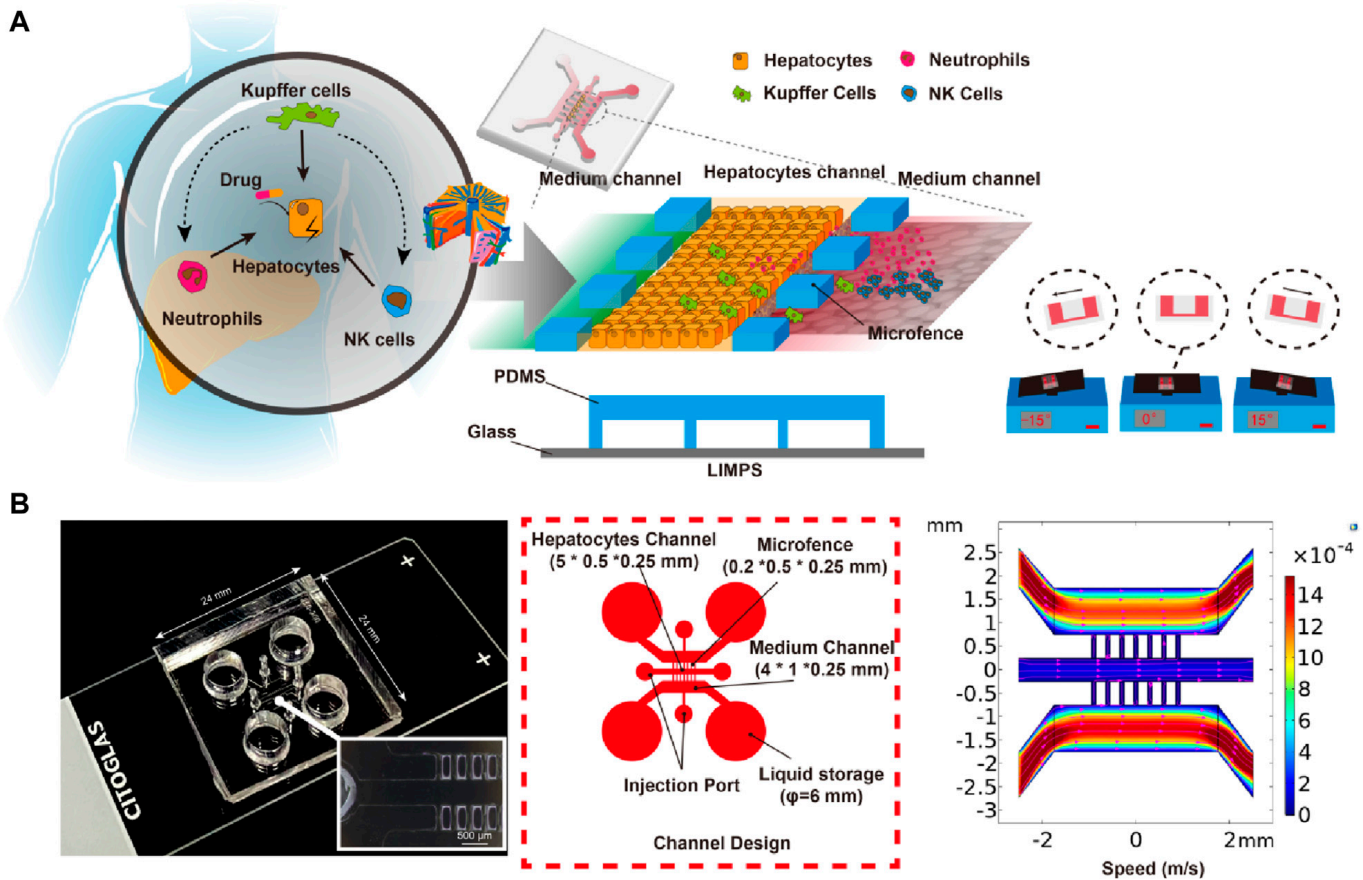
Design and Structure of LIMPS. **(A)** The illustration of the immune-mediated drug-induced liver injury (left), the design of the LIMPS (middle), and the gravity-based pumping (right). **(B)** The real LIMPS and the enlarged view of the micro-fences (left), the channel design (middle), and the microfluidic field inside the LIMPS (right).

In Figure 8, a LIMPS was presented, which was a microfluidic device used to analyze hepatocytes and immune cells. The microfluidic device was fabricated by bonding a polydimethylsiloxane (PDMS) slab with a standard glass slide. The PDMS slab contained three parallel microchannels, the three microchannels through the micro fence (0.2 × 0.5 × 0.25 mm) separation, with a spacing of 0.1 mm between micro fences, where the middle was used to seed hepatocytes, one side microchannel for supplying a blank or TGZ-containing culture medium, and the other side channel for immune cells residence. The microfluidic device was placed on a tilting shaker to generate reciprocating flows in the microchannels that supplies nutrition and oxygen to the cells inside. Analysis of the shear force in the middle microchannel shows a value of 0.1–0.5 dyne/cm^2^, which is in agreement with *in vivo* studies (LeCluyse, et al., 2012a; Rashidi et al., 2016b).

Following the previous studies on immune-mediated liver injury (Deng, et al., 2019; Deng, et al., 2020), we used HepG2, PMA induced THP-1, and DMSO induced HL-60 cells (dHL-60 cells) to represent human hepatocytes, Kupffer cells, and neutrophils, respectively. These cells were chosen based on their sufficient phenotypes relevant to hepatotoxicity and high-quality control standards, resulting in experiments that were more reproducible. Adaptive immune cells, which are only available in a patient’s body and are not obtainable for preclinical screening, were also not included.

This LIMPS offers two distinct advantages. Firstly, it allows for the direct observation of cellular behaviors using an inverted microscope, enabling dynamic monitoring of the liver injury process. Secondly, the immune cell composition can be customized, enabling researchers to identify the functions of each type of immune cell involved in a liver injury process.

### 3.2 Direct observation of the dynamic TGZ-induced liver injury process in LIMPS

#### 3.2.1 Adhesion of dHL60 cells in the LIMPS

According to the established experimental method, we labeled 5 µL of 1 × 10^7^ cells/mL HepG2 cells with green CFSE and 600 µL of 1.5 × 10^6^ cells/mL of dHL-60 cells with yellow Dil (colored by red) and then used a fluorescence microscope to monitor LIMPS lasted for 24 h. The observation showed that dHL-60 cells moved from the side microchannel into the middle microchannel and adhered to the HepG2 cells. We studied the dynamic adhesion process of dHL-60 in the hepatocyte channel with or without TGZ (Figure 1A). In the preliminary experiment, it was observed that a concentration of 15 µM of troglitazone alone did not induce significant toxicity (Supplementary Figure S1). However, it did elicit a notable inflammatory response (Supplementary Figure S2). Consequently, for the adhesion experiment, the concentration of 15 µM troglitazone was employed (Supplementary Figure S1). Figure 1 shows the images of one chip at different time points. By observing the same region, we can see the adhesion process of neutrophils in this region with time increasing. Throughout the process, two kind of cells were co-localized in close contact. To distinguish between these two types of cells, we labeled HepG2 with CFSE (colored by green) and HL-60 with Dil (colored by red) in advance. As shown in Figures 1A, B, in the presence of TGZ, the adhesion of dHL-60 cells initially increases and then decreases, while in the absence of TGZ, the adhesion of dHL-60 cells remains unchanged from 1 to 24 h. This indicates that troglitazone affects the adhesion behavior of dHL-60 cells. To investigate the reasons behind this phenomenon, we observed local HepG2 cells and found that in the presence of TGZ, dHL-60 cells gradually adhere to HepG2 cells. However, at 24 h, the HepG2 cells disappear, leading to a decrease in adhesion. On the other hand, in the absence of TGZ, there is no significant change in the adhesion of dHL-60 cells, and HepG2 cells do not disappear (Figure 1C). This is likely attributed to a severe inflammatory environment. Additionally, it is known that neutrophils undergo cell death after exerting their functions (Mantovani, et al., 2011), which could contribute to the decrease in the number of dHL-60 cells. Moreover, the disappearance and shedding of HepG2 cells lead to a decrease in the number of dHL-60 cells adhering to HepG2 cells.

#### 3.2.2 Polarization of the THP-1 cells

We loaded HepG2 cells (5 μL, 1 × 10^7^ cells/mL) or not into LIMPS, added THP-1 cells (50 μL, 3 × 10^6^ cells/mL) on both sides of LIMPS, waited for cell adhesion and pre-treated with TGZ for 48 h, then added dHL-60 cells (600 μL, 1.5 × 10^6^ cells/mL) to LIMPS for 24 h. We observed that THP-1 cells have different shapes in different experimental groups in the LIMPS. Figure 7A shows the relationship between circularity values and cell shape. To assess the degree of polarization in macrophages, we employed circularity calculations, with smaller circularity values reflecting a higher level of polarization. The extent of polarization was quantified by enumerating the number (or proportion) of circularity ranges, by the established protocol outlined in the study by Sheppe, et al. (2018). Specifically, we quantified the occurrence of polarization, by tallying the number of macrophages exhibiting this polarization (defined as a circularity range of 0–0.2). To further elucidate the relationship between circularity and cell morphology, we presented illustrative images of cells with circularity values ranging from 0 to 0.2 in Supplementary Figure S3. Utilizing ImageJ software, we calculated the circularity values of THP-1 cells within the LIMPS system and enumerated the cells exhibiting distinct circularity values. The green coloration in Figure 7B represents CD163 immunofluorescence staining. In this experiment, we initially utilized PBS to thoroughly wash the side channels of the chip. The majority of dHL-60 cells present in these side channels did not adhere to the bottom surface of the chip due to their nature as suspended cells, which flow with the fluid. Even if a minor quantity of dHL-60 cells adhered to THP-1 cells, we considered them as a single entity for counting purposes. Consequently, we calculated the percentage of THP-1 cells exhibiting a low circularity (ranging from 0 to 0.2). As shown in Figure 7B, in the presence of HepG2 cells, the troglitazone or dHL-60 cells alone resulted in the activation of THP-1 cells (0<circularity<0.2); the co-existence of troglitazone and dHL-60 cells also resulted in the activation of 60% of THP-1 cells (in the side channel). Activated THP-1 cells can exhibit pro-inflammatory M1 and anti-inflammatory M2 states, characterized by the CD86 and CD163 biomarkers, respectively. Figure 3C demonstrates that in the presence of HepG2 cells, the addition of troglitazone and dHL-60 cells results in a decrease in CD163 expression and an increase in CD86 expression, as captured in the side channel images. This finding suggests that both troglitazone and neutrophils contribute to the modulation of the proinflammatory and anti-inflammatory states of liver macrophages.

As illustrated in Figure 7D, in the absence of HepG2 cells, there is a relatively high incidence of THP-1 cells displaying reduced a low circularity, reaching 40%. However, when HepG2 cells are introduced, the percentage of THP-1 cells with diminished a low circularity drops to approximately 25%, indicating a potential inhibitory impact of HepG2 on the polarization of THP-1 cells. Moreover, upon the addition of TGZ, there is a notable increase in the proportion of THP-1 cells with a low circularity, signifying the reversal of HepG2’s inhibitory effect on THP-1 cell polarization by TGZ. Therefore, it indicates that TGZ has a strong ability to activate polarization in THP-1 cells. These results indicate that multi-cellular participation in the presence of TGZ stimulates inflammation in the liver and aggravates liver damage.

### 3.3 Investigation of TGZ-induced liver injury in the customized immune microenvironments in LIMPS

#### 3.3.1 The effect of the THP-1 cells on the hepatotoxicity due to TGZ

Kupffer cells are a type of macrophage found in the liver. Under normal physiological conditions, Kupffer cells make up approximately 12% of the liver’s cellular composition. However, during times of inflammation, Kupffer cells have been found to accumulate in the liver (Meng, et al., 2020). In our study, we sought to examine the relationship between Kupffer cell count and liver injury in the presence of TGZ.

After the adhesion of HepG2 cells (5 μL, 1 × 10^7^ cells/mL), varying amounts of THP-1 cells were introduced followed by the perfusion of LIMPS with a concentration of 15 µM TGZ for 48 h. Figure 4A demonstrates that when the number of HepG2 cells, as assessed by the area of GFP pixels, remained constant in the presence of THP-1 cells from up to 5 × 10^5^ without TGZ treatment or up to 2.5 × 10^5^ with TGZ treatment. However, HepG2 cells were reduced by nearly 40% in the presence of 5 × 10^5^ THP-1 cells and treated with 15 µM TGZ. Additionally, Figure 4B illustrates that THP-1 changes the morphology of surviving HepG2 cells from a polygonal to a round shape in the presence of 15 µM TGZ, indicating considerable immune stress on HepG2 cells. Notably, the HepG2 cells are transfected with GFP via a strong transcription factor. Declined fluorescence of GFP indicates a halt in transcription or deactivation of the endoplasmic reticulum. Compared to the markers of cell proliferation rate, ROS quantity, and RNA changes indicating mild liver injury, declined GFP expression is a better indicator of severe liver cell damage and the potential for liver failure in clinical settings.

At the endpoint of the experiment, we detected the content of inflammatory factors in LIMPS. In Figure 4C, the increase in the number of THP-1 cells is associated with a corresponding increase in the secretion of inflammatory factors interleukin-1β (IL-1β), tumor necrosis factor-α (TNF-α), and interleukin-6 (IL-6) and anti-inflammatory factor interleukin-10 (IL-10) in the LIMPS. A significant difference in IL-1β secretion is observed with or without 15 µM of TGZ when the number of THP-1 cells is 5 × 10^4^. Moreover, the exponential growth of TNF-α aligns with the secretion tendency of THP-1 cells. Similarly, a significant difference in IL-6 secretion is observed when the number of THP-1 cells is 5 × 10^5^. Meanwhile anti-inflammatory factor IL-10 decreased. These findings suggest that escalation of inflammatory factor levels occur preceding liver injury in the LIMPS that occurs at 5 × 10^5^ THP-1 cells. Additionally, when the number of THP-1 cells is 5 × 10^5^, all four inflammatory factors in the LIMPS exhibit significant differences in comparison to the control.

The study suggests that Kupffer cells do not usually affect the hepatotoxicity of TGZ under normal physiological conditions. However, increased levels of Kupffer cells in the liver due to inflammation can intensify the toxic effects of TGZ. TGZ also reduces the levels of IL-10 anti-inflammatory factors and increases the levels of inflammatory factors, which in turn lead to the recruitment of more immune cells from the peripheral blood and further exacerbate liver injury.

#### 3.3.2 The effect of the dHL-60 cells on the hepatotoxicity due to TGZ

We perfused a LIMPS (with HepG2 cells, 5 μL, 1 × 10^7^ cells/mL) with 15 µM of TGZ for 48 h and then introduced dHL-60 cells (600 μL, 1.5 × 10^6^ cells/mL) incubating for an additional 24 h. Using Calcein AM (green), PI (red), and HOE33342 (blue), we co-stained the HepG2 cells and evaluated live cell proportion based on the ratio of live cell area to the total cell area (Figure 2A; Supplementary Figure S10). Our results showed that neither TGZ nor dHL-60 alone had any impact on the live cell proportion, but when co-presented, we observed a significant decrease in the live cell proportion, indicating liver injury (Figure 2B). We calculated the area of green fluorescence to measure the absolute quantity of the live HepG2 cells and found that only 45% of the cells remained alive in the presence of both TGZ and dHL-60, indicating severe cell death (Figure 2C). The level of severity reached a point where the nucleus of HepG2 cells could no longer be stained by HOE33342. At the end of the experiment, we used qPCR to measure mRNA levels in the dHL-60 cells in the LIMPS and found a significant upregulation of relevant cytokines (e.g., TNF-α, IL-1β), chemotaxis factors [e.g., Macrophage inflammatory protein-2 (MIP-2), Monocyte chemotactic protein-1 (MCP-1)], and adhesion factors [e.g., Macrophage-1 antigen (MAC-1), Intercellular cell adhesion molecule-1 (ICAM-1)] (Figure 2D), further revealing the synergy between dHL-60 and TGZ in eliciting hepatotoxicity. Regrettably, no alterations were detected in the myeloperoxidase gene (MPO), potentially attributed to the inherent limited expression of the MPO gene in dHL-60 cells. It is noteworthy that MIP-2, an effective neutrophil chemoattractant and activator, is highly expressed, indicating that TGZ not only activates neutrophils to exacerbate liver injury, but also continues to attract more neutrophils to gather locally and trigger subsequent liver damage, potentially leading to acute liver failure in clinical settings.

#### 3.3.3 The effect of the NK-92 cells on the hepatotoxicity due to TGZ

Natural killer cells (NK cells) are important members of the immune system and play a significant role in recognizing and clearing foreign invaders. We therefore investigated the toxicity of TGZ in the presence of NK-92 cells. The methodology employed in this study was as per the earlier description. A LIMPS (with HepG2 cells, 5 μL, 1 × 10^7^ cells/mL) was perfused with a concentration of 15 µM TGZ for 48 h, followed by the introduction of NK-92 cells (600 μL, 1.5 × 10^6^ cells/mL) and subsequent incubation for an additional 24 h. Firstly, we found that NK-92 cells alone exhibited cytotoxicity against HepG2 cells (Figures 3A, B, the proportion of area calculated by Green area/(Green area + Red area)), which is consistent with the reported elsewhere (Ayuso, et al., 2021). Surprisingly, this cytotoxicity did not increase or remain the same with the addition of TGZ, but instead decreased (Figure 3B, +NK-92/-TGZ vs. + NK-92/+TGZ). Therefore, we separately tested the impact of TGZ on NK-92 cells (Figure 3C) and found that 15 μM of TGZ inhibited NK-92 cell proliferation by approximately 50% (cell activity measured by CCK-8 method). There are no reports on the effect of TGZ on NK cells previously. However, a recent study (Kobayashi, et al., 2020) demonstrated that peroxisome proliferator-activated receptor γ (PPARγ) agonists can reduce the mitochondrial membrane potential of NK cells, thereby inhibiting their function. It is worth noting that TGZ is a PPARγ agonist, suggesting that it may have an inhibitory effect on NK cells reprogramming *in vivo*. That meant NK cells should have no significant contribution to troglitazone-induced hepatotoxicity. In addition, we observed NK-92 cells migrate into hepatic channels, yet our observations indicate that they do not adhere to hepatocytes, allowing for their facile removal from the chip through PBS washing. The underlying cause for NK-92 cells to eliminate HepG2 cells may involve the cytotoxic mechanisms inherent to NK-92 cells themselves. The above results further demonstrate that the LIMPS can not only reflect the interaction between one type of cell and drugs but also the interaction between multiple cells and drugs.

#### 3.3.4 The effect of the co-presence of THP-1 and dHL-60 cells on the hepatotoxicity due to TGZ

We conducted a study to examine the hepatotoxic effects of TGZ in the presence of THP-1 and dHL-60 cells. Employing the experimental procedure as outlined, THP-1 (50 μL, 3 × 10^6^ cells/mL) and dHL-60 cells (600 μL, 1.5 × 10^6^ cells/mL) were introduced into LIMPS. Figure 5A demonstrated that the presence of both THP-1 and dHL-60 cells resulted in significant damage to the LIMPS. Notably, the extent of liver damage caused by immune cells combination (55%) was greater than the sum of that caused by each cell type separately (35%), indicating that THP-1 and dHL-60 cells work synergistically, thereby intensifying the severity of the injury. We detected the levels of inflammatory factors in LIMPS at the endpoint of the experiment, as demonstrated in Figure 5B, this synergistic interaction was further reflected in the cytokine secretion levels, with pro-inflammatory cytokines (IL-1β and TNF-α) being higher, whereas anti-inflammatory cytokine (IL-10) was lower in the immune cell combination group compared to the sum of each cell group independently. These findings suggest that macrophages and neutrophils complement each other in liver and trigger a more strong immune-inflammatory response.

Finally, we examined the hepatotoxicity of the control drugs rosiglitazone and pioglitazone using the LIMPS. We employed the same experimental protocol and utilized a concentration that was 2.5-fold the Cmax as the experimental concentration. The results revealed that at this concentration, pioglitazone and rosiglitazone exhibited minimal immune cell interference in terms of hepatotoxicity, whereas troglitazone exhibited significant variations (consistent with previous experiments). This suggests that pioglitazone and rosiglitazone have minimal immune hepatotoxicity, or that higher concentrations are required for immune hepatotoxicity to manifest.

## 4 Discussions

In this study, we aimed to develop a liver-immune-microphysiological-system (LIMPS) to investigate immune-mediated liver damage. The use of human-originated cells in LIMPS presents a significant advantage as it can lower or eliminate the need for animal experimentation in pre-screening drug-induced liver injury during drug development. Science News reported in January 2023 that the FDA no longer requires animal testing before human drug trials, which has spurred a new wave of research aimed at developing alternative models. Microphysiological systems (MPS) have been at the center of these efforts, but few studies have focused on the development of MPS with an immune microenvironment.

In this study, we present a novel LIMPS and evaluate its potential using TGZ as a model drug. Our results from LIMPS enabled several new discoveries that provide a more profound understanding of TGZ-induced liver failure in clinical settings. The power of LIMPS becomes evident from another perspective as well. Early research results (Kim et al., 2017a; Granitzny, et al., 2017) showed that the supernatant of co-culturing troglitazone with liver cells can lead to immune cell activation. Due to their non-contact system, which is different from the *in vivo* situation, it is not possible to directly observe how immune cells work. However, our study can see the direct contact between immune cells, LIMPS can visually observe the activation and adhesion of immune cells to aid in the occurrence of toxicity. They did not prove that activated immune cells can cause liver cell damage, while we have demonstrated on LIMPS that liver cells can be damaged in the presence of TGZ and immune cells. In addition, one studies (Novac, et al., 2022) based on co-culture of immune cells and liver cells found that troglitazone toxic concentration was 54 μM. Although the immune cells were in contact with liver cells, the type of immune cells was single, and they did not evaluate the function of multiple immune cells. Our LIMPS showed a lower toxicity concentration of troglitazone, and we found that under the action of the combination of two immune cells, the toxicity of troglitazone was enhanced. In traditional *in vitro* models (Rachek, et al., 2009b; Shoichiro et al., 2003), hepatocyte death was observed only when the TGZ concentration exceeds 50 μM, which is much higher than the maximum therapeutic dose of 3–6.25 μM for humans (Chojkier, 2005). However, in LIMPS, we discovered hepatocyte death even at a concentration of 15 μM, which is closest to the clinical exposure. As time went on, the damage became more and more severe, we found that 75% of the HepG2 cells were damaged and disappeared at the end of 144 h (Supplementary Figure S4). Other research concerning immune hepatotoxicity (Kim et al., 2017a; Granitzny, et al., 2017; Novac, et al., 2022), the experimental time is between 48 and 96 h. In our experiment, differences between the experimental group and control group emerged at 48 h. Therefore, we selected 48 h as the time point for our study. Additionally, we speculate that if choosing 144 h as the time point, even lower concentrations of TGZ could potentially induce damage. An obvious advantage of LIMPS is the ability to provide multiple influencing factors, such as the quantity and types of immune cells, as well as the mechanical microenvironment experienced by various cells. We can observe that the fluid environment in the side channel is different from that in the middle channel (Supplementary Figure S5), providing different fluid environments for different cells, aiming to simulate the situation where neutrophils are flowing while liver parenchymal cells can withstand lower shear forces. In a macro perspective, all cells would be exposed to the same fluid environment the hepatic sinusoids and hence the environment should not be different. However in a microscopic perspective, different cells are in a different fluid environment. References (LeCluyse, et al., 2012b; Rashidi, et al., 2016b) indicate that liver cells experience shear forces several orders of magnitude lower than those in the hepatic sinusoid vein or artery. Additionally, in the study (Ferrari, et al., 2023), endothelial cells have formed a circular channels. Therefore, the liver cells are only subjected to minimal flow rates and shear forces around them. Our system has a shear force approximately 100 times lower in the middle channel compared to the two side channels (Supplementary Figure S5), which is similar to the situation described in the literature where the shear force on liver cells is lower than that on the hepatic sinus vein or artery (LeCluyse et al., 2012a; Rashidi et al., 2016b). Since the purpose of this study is to investigate the role of three kind of immune cells in the liver injury caused by troglitazone, we did not incorporated endothelial cells. Our system has certain limitations in simulating the precise structure of hepatic sinusoids. Under *in vivo* physiological conditions, Kupffer cells are not completely in direct contact with liver cells. Most of the Kupffer cell bodies are located in the hepatic sinusoids (special capillaries in the liver), and some of the pseudopodia of the cells penetrate the fenestra and extend into the perisinusoidal space, making contact with liver cells. This type of microstructure is too difficult to completely simulate *in vitro* until now.

Oda et al. (2016) reported that genes such as MCP-1, IL-1β, and IL-8 were upregulated in dHL-60 cells following stimulation by the supernatant of HepG2 cells incubated with 100 μM TGZ. However, they did not investigate the effects of dHL-60 cells on liver cells. In our study, we not only detected the inflammatory factors in the LIMPS but also observed the adhesion of dHL-60 cells to HepG2 cells, which led to the death of HepG2 cells in the presence of TGZ. This observation sheds light on the potential mechanisms behind TGZ-induced liver failure in clinical settings.

Our observations indicate that the presence of TGZ potentiates the crosstalk between macrophages and neutrophils. As evidenced by the changes in inflammatory cytokines presented in Figure 5B, the concurrent addition of macrophages and neutrophils led to an increase in inflammatory cytokines, exceeding the levels observed when either cell type was added alone. This suggests the existence of an interactive relationship between macrophages and neutrophils. Furthermore, the inflammatory cytokines in the drug-treated group were more pronounced than those in the control group (not shown in the main text but included in the Supplementary Figure S8), indicating that the presence of TGZ enhances the interaction between macrophages and neutrophils. Additionally, morphological analysis of macrophages (depicted in Figure 7B) revealed that the drug-treated group exhibited stronger effects in the presence of HL60 cells compared to the untreated group, further corroborating the enhanced crosstalk between macrophages and neutrophils in the presence of TGZ. Although we do not have direct experimental evidence to support the specific mechanism, we hypothesize that pretreatment with TGZ may induce stress in HepG2 cells, leading to the polarization of THP-1 cells. The addition of HL-60 cells further amplifies this polarization, thereby enhancing the formation of a proinflammatory environment.

Using LIMPS, we also investigated the role of macrophages in TGZ-induced liver injury. THP-1 cells were introduced into adjacent channels. According to our observations, after 48 h, about 2% of the THP-1 cells fell into the intermediate channel since THP-1 cells have a different shape compared to HepG2 cells. By monitoring the inflammatory factors in the LIMPS, it was found that macrophages could synergize with neutrophils to release more IL-1β and TNF-α under the influence of TGZ. The anti-inflammatory factor IL-10 was reduced under this condition, indicating enhanced interactions between immune cells only under the influence of TGZ. In the absence of HepG2 cells, the activation of THP-1 cells is relatively high. However, the introduction of HepG2 cells results in a decrease in the activation of THP-1 cells, suggesting that HepG2 cells also play a role in this immune modulation.

Traditional cell experiments have been limited to the study of dose-toxicity relationships, while the temporal-spatial dynamics of cells are often difficult to characterize. Our LIMPS system enables the observation of the adhesion, chemotaxis, and other processes of neutrophils. By localizing hepatocytes, macrophages, and neutrophils within a chip, we observed morphological changes in macrophages. The significance of interactions between different immune cells and between immune cells, hepatocytes, and drugs is emphasized in this study. It should be noted that cell lines such as HepG2 cells can only exhibit partial functionality of the liver and cannot fully represent the true conditions *in vivo*. Therefore, it is necessary to explore better cell sources in future experiments.

Our findings also showed that the inflammation state of the circulatory system could play an important role in TGZ-induced liver injury. TGZ-induced severe hepatotoxicity requires a high concentration of dHL-60 with 1 × 10^7^ cells/mL, which is above the upper limit of the normal range of neutrophils in the blood (0.63 × 10^7^ cells/mL), indicating that the deposition of immune cells in the body could be a triggering for TGZ-induced hepatotoxicity. This underscores the need for restricting the use of specific populations for this kind of hepatotoxicity drugs. Our results observed an increase in the expression of some inflammation genes, indicating the importance of immune cells in the *in vitro* system. However, the mechanisms of neutrophil damage are diverse, including proteases, reactive oxygen species, etc. (Jaeschke and Smitht, 1997; Hartmut, 2000). The specific mechanism may not be known yet, and it deserves in-depth research to understand what neutrophils interact with liver cells in the future. We need to establish a proper evaluation method to predict drug-induced liver injury during drug development. Overall, the change in the immune microenvironment is the main cause of sensitivity changes in TGZ-induced liver toxicity, and it is important to incorporate various immune cells into the LIMPS for drug safety evaluation.

## 5 Conclusion

We have developed a liver microphysiological system that incorporates a controllable immune microenvironment. The fabrication of the microphysiological system is simple and standardizable. In addition, we have established a protocol that enables us to decipher immune-mediated drug-induced liver injury. Using our protocol, we have successfully identified the immuno-hepatotoxicity of troglitazone, which represents a significant breakthrough in the toxicity evaluation of drugs. This methodology is versatile and can be applied to screen immuno-hepatotoxicity during the development of new drugs, which may reduce the use or eventually eliminate the need of animal studies.

## Data Availability

The raw data supporting the conclusion of this article will be made available by the authors, without undue reservation.
