# Supplementary material for: Assessing immune hepatotoxicity of troglitazone with a versatile liver-immune-microphysiological-system
Source: Front Pharmacol. 2024 May 30;15:1335836. doi: 10.3389/fphar.2024.1335836 (PMC11169855; doi:10.3389/fphar.2024.1335836)
Supplement: Supplementary file 1 [file DataSheet1.docx]

Supplementary Material

# Supplementary Data

We compared the toxicity of troglitazone and rosiglitazone in LIMPS and LMPS. As we konw lactate dehydrogenase (LDH), alanine aminotransferase (ALT), and aspartate aminotransferase (AST) were released into the extracellular space when there is cell damage, the rupture of the cell membrane or changes in permeability.The drug toxicity is determined by detecting the content of LDH, ALT, and AST in the culture medium.

We established different experimental groups with concentrations of 0, 0.05x, 0.5x, 2.5x, 5x, 10x, and 25x of the therapeutic concentrations detected in patient serum (C_max_). It is known from the literature that the therapeutic concentrations detected in patient serum of troglitazone is reported to be 6 μM, and the experimental group concentrations are 0 μM, 0.3 μM, 3 μM, 15 μM, 30 μM, 60 μM, and 150 μM. The therapeutic concentrations detected in patient serum of rosiglitazone is reported to be 1 μM, and the experimental group concentrations are 0 μM, 0.1 μM, 1 μM, 5 μM, 10 μM, 20 μM, and 50 μM.

As shown in Figure S1, in the LIMPS group, the levels of LDH, ALT, and AST increased with the increasing concentration of troglitazone. ALT exhibits significant differences at 2.5x of therapeutic serum levels, while AST and LDH exhibit significant differences at 10x of therapeutic serum levels. In the LMPS group, there are no significant differences in the levels of LDH, ALT, and AST.

We further compared the toxicological differences between the LIMPS and LMPS at the same drug concentration. At 2.5x of therapeutic serum levels of troglitazone, there are significant differences in the levels of ALT and LDH between the LIMPS group and the LMPS group. AST shows significant differences at 10x of therapeutic serum levels.There are no significant differences in the levels of ALT and AST with the increasing concentration of rosiglitazone. However, the level of LDH significantly increases at 20x and 50x of therapeutic serum levels. There are no significant differences in the liver toxicity between the LIMPS and LMPS at the same drug concentration. These results indicate that the inclusion of immune cells leads to an increased liver toxicity of troglitazone.

We loaded HepG2 cells (5 µL, 1×10^7^ cells/mL) or not into LIMPS, added THP-1 cells (200 µL, 3×10^6^ cells/mL) on both sides of LIMPS, waited for cell adhesion and pre-treated with TGZ for 48 hours, then added dHL-60 cells (600 µL, 1.5×10^6^ cells/mL) to LIMPS for 24 hours.We furthercompared the total area of residual cells in LIMPS under different concentrations of troglitazone(Supplementary Figure 2), and it can be observed that there is a significant difference in the green fluorescence area when the concentration of troglitazone is 15 μM.

we assessed the inflammatory response in LIMPS under different concentrations of troglitazone stimulation. The results demonstrated that a concentration of 15 μM of troglitazone elicited the highest inflammatory response, with the greatest release of the IL-1β. Conversely, following stimulation with 30 μM of troglitazone, the IL-1β content decreased, potentially indicating an inhibitory effect of higher concentrations of troglitazone on immune cells. In conclusion, we have observed the potential of the LIMPS for detecting drug toxicity in our preliminary study.

# Supplementary Figures and Tables

## Supplementary Figures


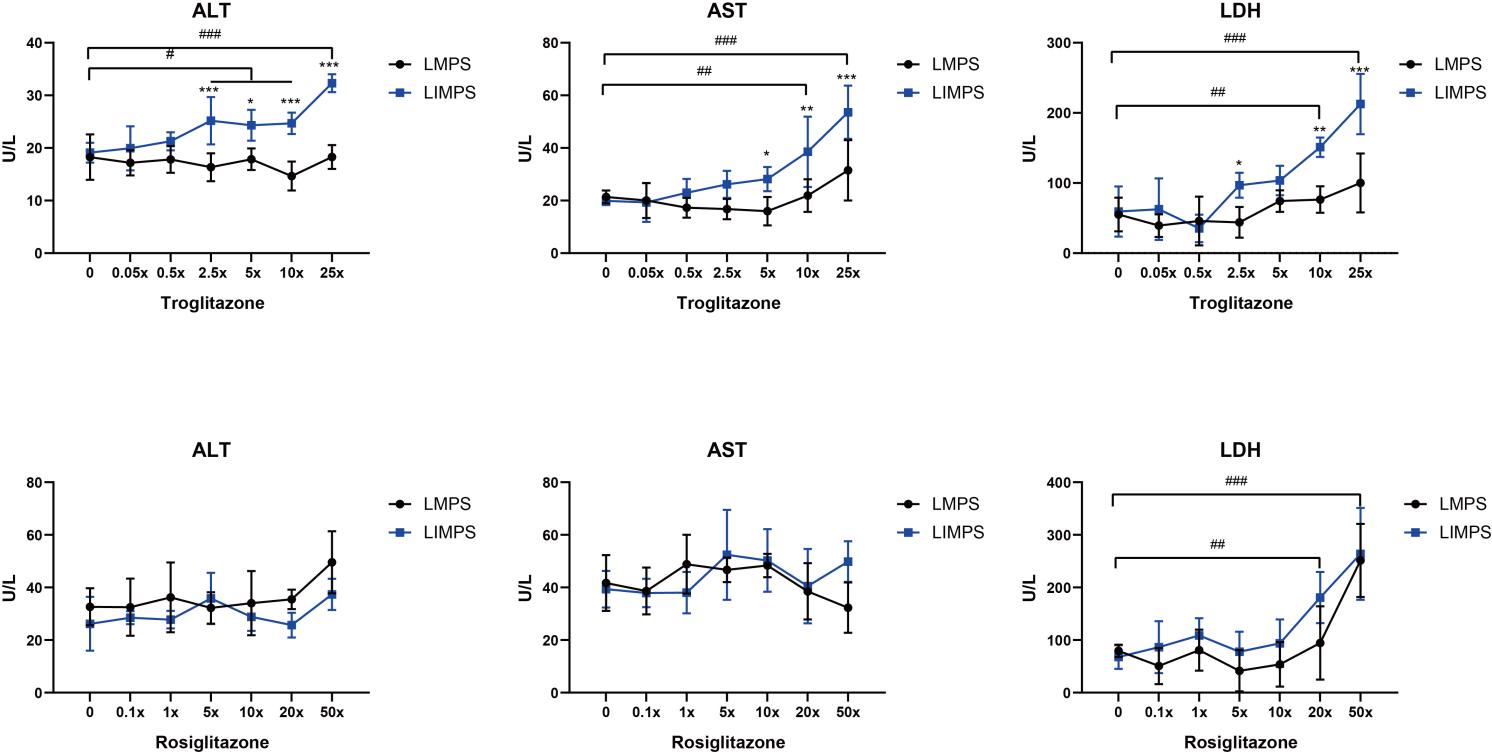


**Supplementary Figure 1.** The levels of ALT, AST, and LDH in LIMPS and LMPS treated with different concentrations of troglitazone and rosiglitazone.（n=3，*p<0.05，**p<0.01，***p<0.001）


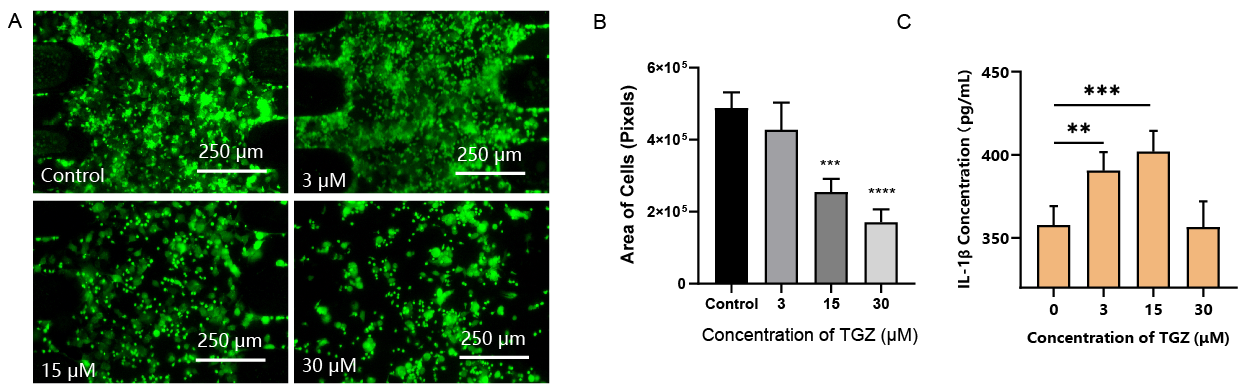


**Supplementary Figure 2.** (A) shows the activity staining of the LIMPS and the green fluorescence of the intermediate channel. (B) The statistical plot of fluorescence area in A. (C) IL-1β in medium Changes in LIMPS.（n=3，*p<0.05，**p<0.01，***p<0.001）


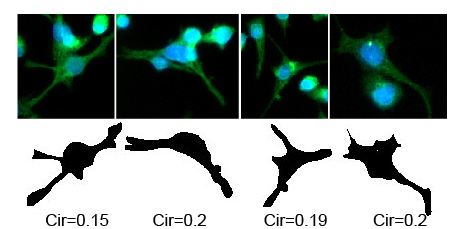


**Supplementary Figure 3.** TheTHP-1 cells of circularity from 0 to 0.2.


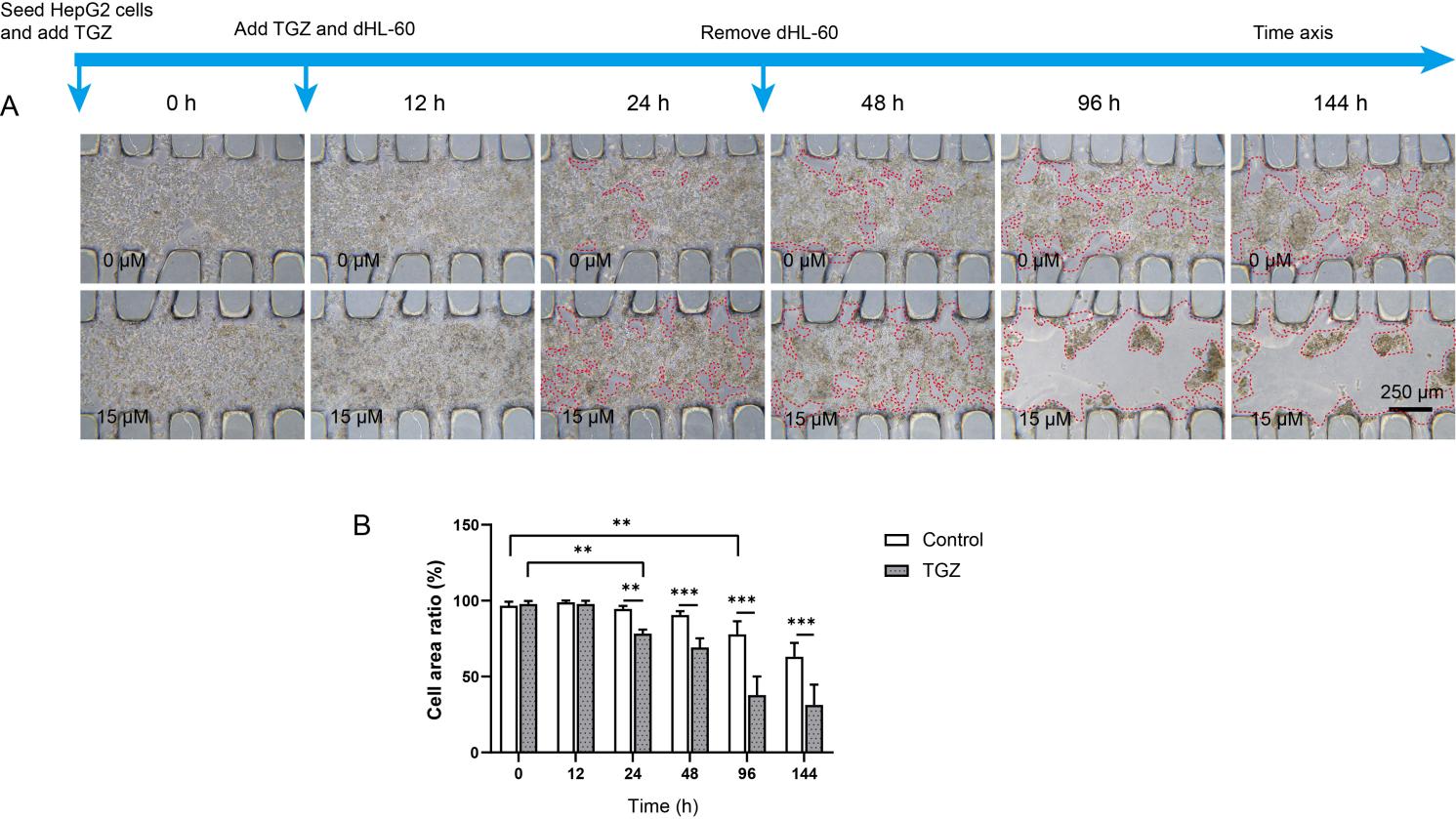


**Supplementary Figure 4.** Light field image of the liver cell region of the liver chip treated with triglitazone over time (A) Light field image of the liver cell region of the LIMPS over time (B) Statistics of the proportion of A's cell area.（n=3，*p<0.05，**p<0.01，***p<0.001）


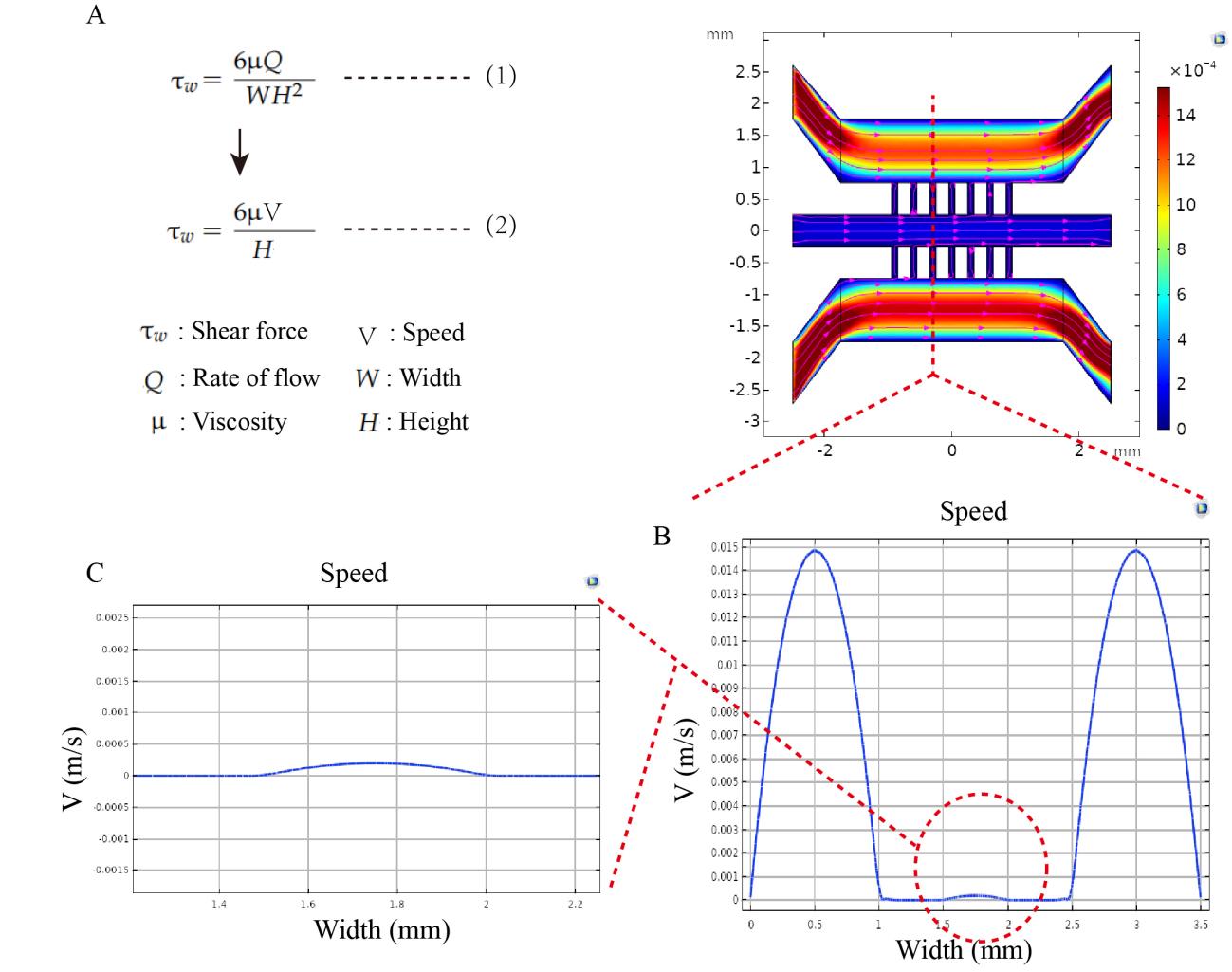


**Supplementary Figure 5.** Fluid simulation of the LIMPS. (A) The shear force formula and flow velocity field on the bottom surface of the LIMPS. (B) The flow velocity at the cross-section of the channel. (C) The flow velocity at the cross-section of the central microchannel.


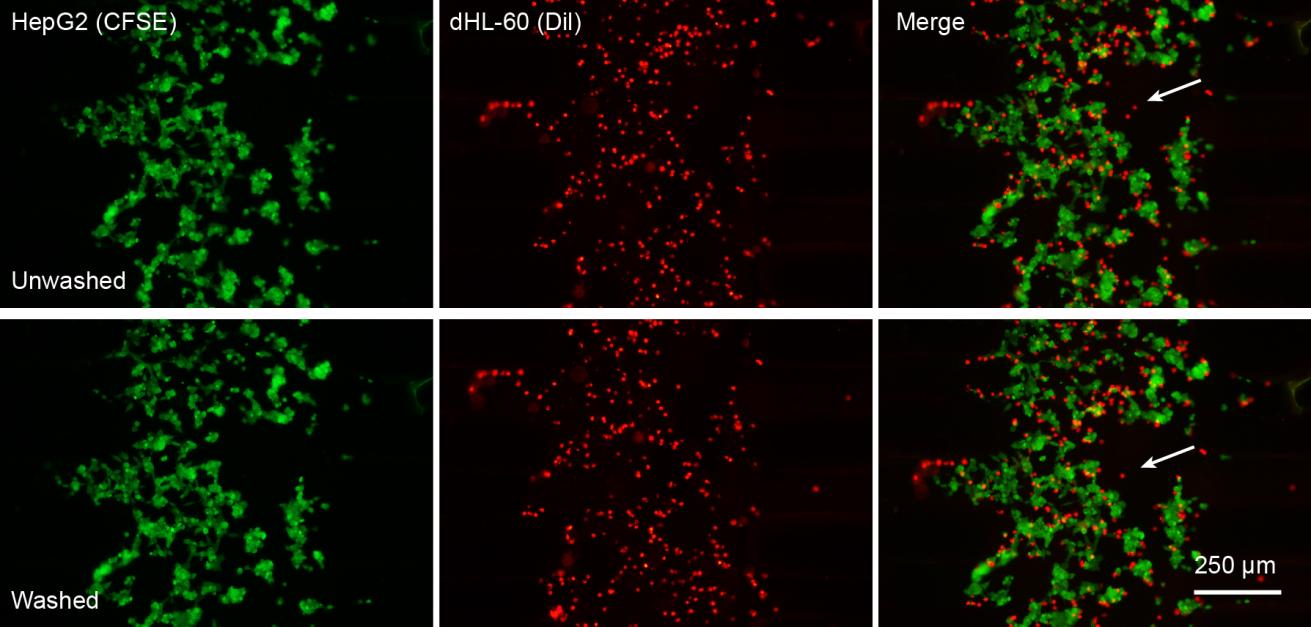


**Supplementary Figure 6.** Adhesion of dHL-60 cells before and after washing the channel.


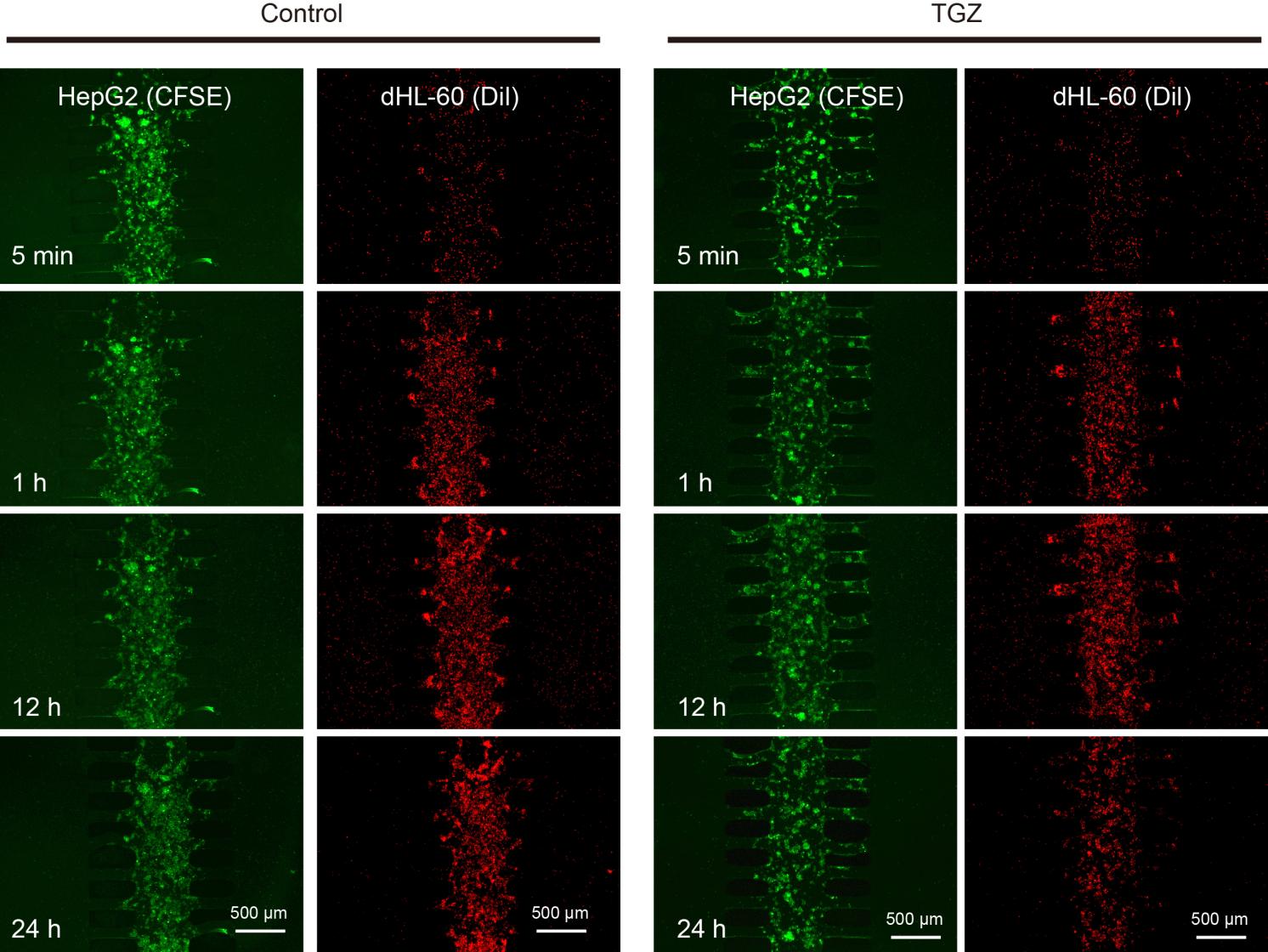


**Supplementary Figure 7.** Adhesion of dHL-60 cells on the HepG2 cells under different circumstances (4x image).


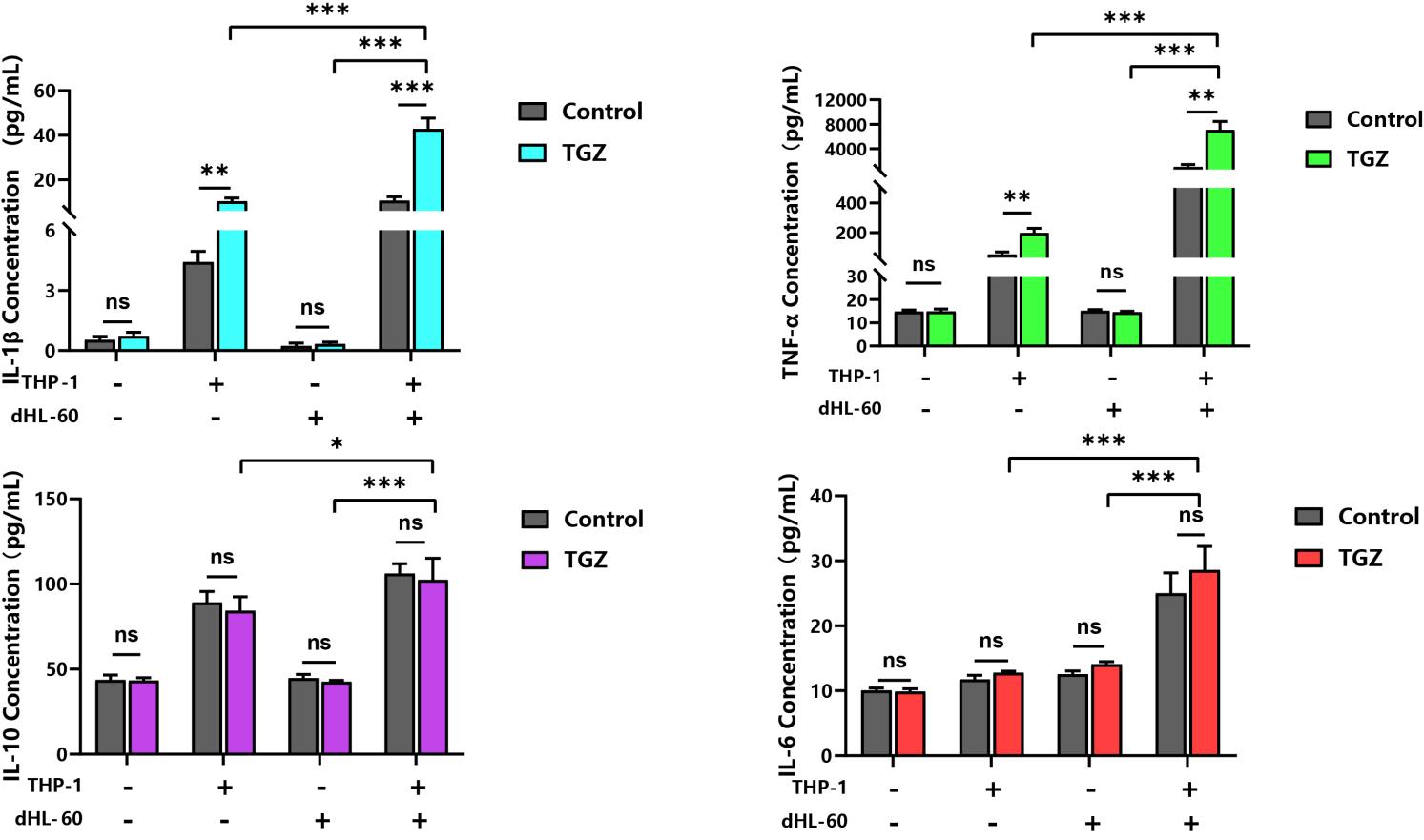


**Supplementary Figure 8.** The supplement to Figure 7 shows secretion of the factors (IL-1β, TNF-α, IL-6, IL-10) under the different circumstances (including drug group and control group). (n = 3 , * p < 0.05,** p < 0.01,*** p < 0.001)

**
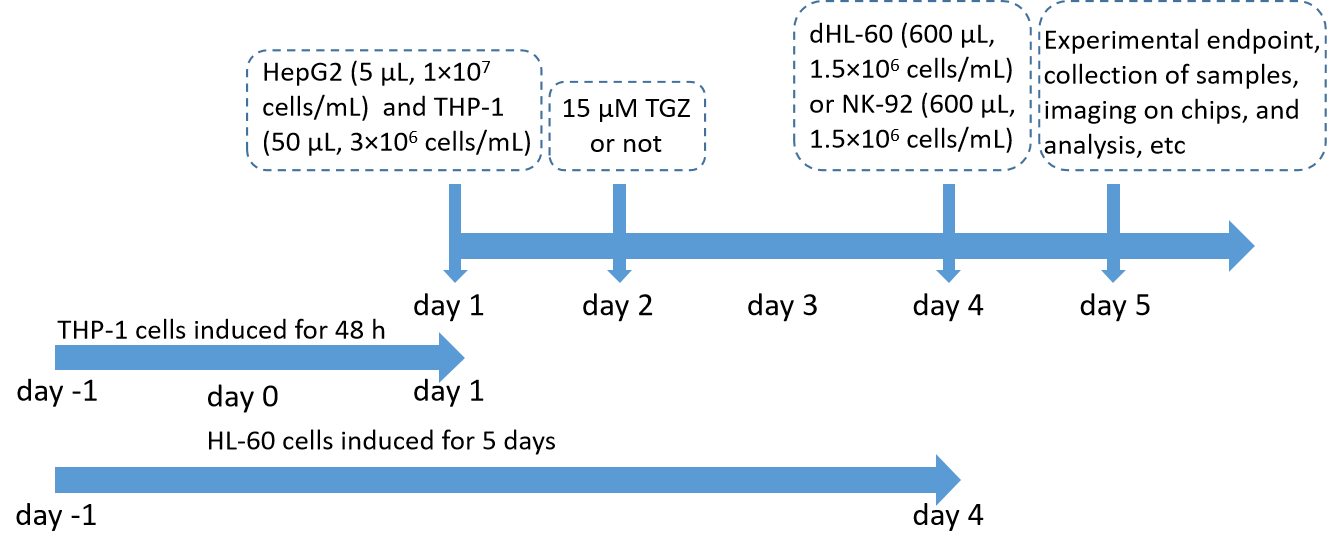
**

**Supplementary Figure 9.** A schematic diagram to depict the timeline of the overall experiment procedure.


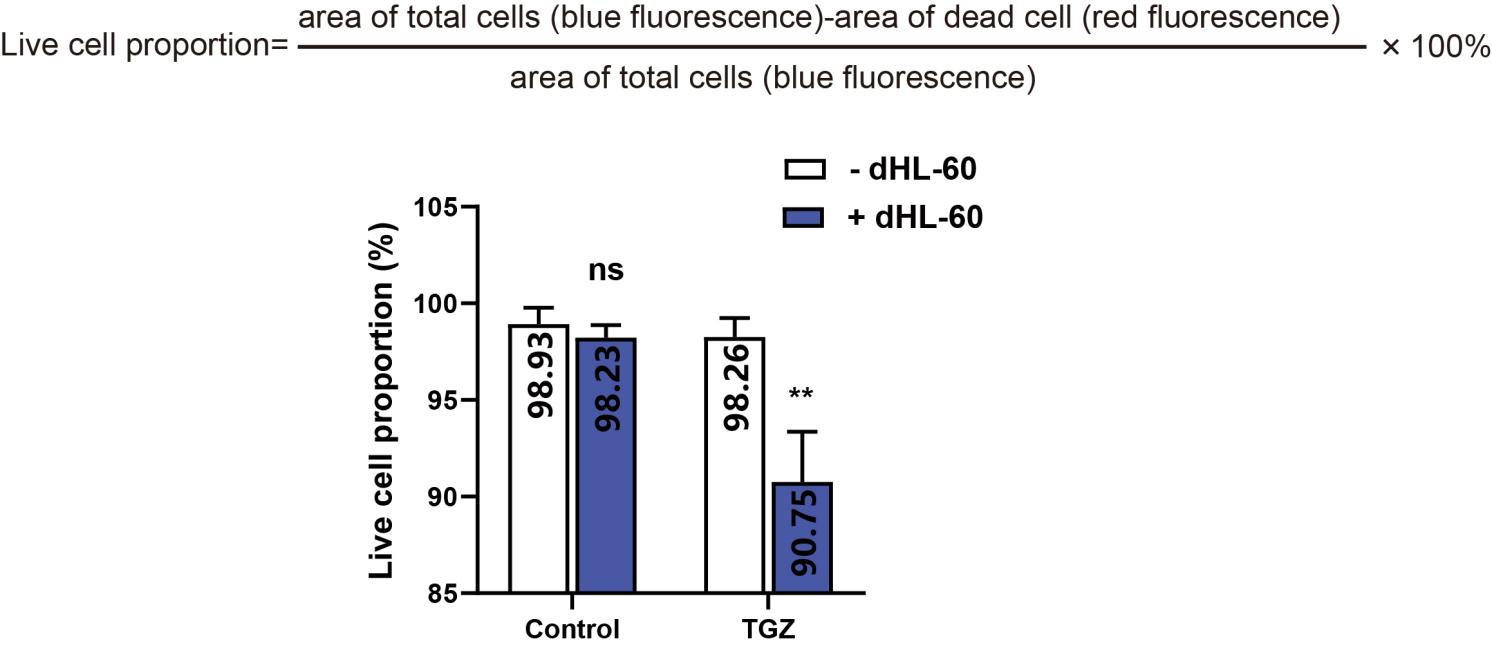


**Supplementary Figure 10.** The supplement to Figure 5 shows the calculation of live cell proportion.
